# Gallium-Based Liquid Metal Microcapsule Composites: A Synergistic Solution to the Challenges of Heat Dissipation, Electrical Insulation and Electromagnetic Shielding

**DOI:** 10.3390/molecules31173009

**Published:** 2026-08-27

**Authors:** Jie Bai, Cong Gu, Shunfeng Sang, Zixin Zhang, Xiao Zhou, Junyu Liu, Shu Yang, Qiang Wang

**Affiliations:** 1 Chongqing Key Laboratory of Green Aviation Energy and Power, Chongqing Jiaotong University, Chongqing 400074, China; baij@cqjtu.edu.cn (J.B.);; 2 School of Mechatronics and Vehicle Engineering, Chongqing Jiaotong University, Chongqing 400074, China; 3 Key Laboratory of Low-Grade Energy Utilization Technologies and Systems, Ministry of Education, Chongqing University, Chongqing 400044, China

**Keywords:** liquid metals, microcapacitor structures, thermal conductivity, insulation, electromagnetic shielding effectiveness

## Abstract

The miniaturization and high-frequency development of electronic devices demand advanced packaging materials that simultaneously possess improved thermal conductivity, notable electromagnetic interference shielding effectiveness, and reliable electrical insulation. Although liquid metals exhibit superior thermal and electrical conductivity, their potential leakage may cause environmental contamination and corrosion of electronic components. Aiming at this dilemma, we designed novel liquid metal microcapsule composites. The composite features Ga-In alloy droplets encapsulated within poly(urea-formaldehyde) shells, which effectively harness the advantageous properties of liquid metals while preventing leakage risks and providing electrical insulation through the polymeric coating. Four microcapsules with different particle sizes (10.6, 9.8, 8.6, and 7.6 μm) were prepared, and LMMCS were synthesized. Comprehensive characterization revealed that, the composite containing 60 wt% of 9.8 μm microcapsules exhibited optimal thermal conductivity (0.527 W·m^−1^·K^−1^). The 60 wt% 7.6 μm composite demonstrated the highest volume resistivity (8.99 × 10^11^ Ω·cm). EMI shielding effectiveness increased with microcapsule loading, while showing non-monotonic diameter dependence at a fixed 60 wt% concentration. Beyond conventional shielding mechanisms, we developed a microcapacitor model that elucidates the size-dependent interfacial polarization and multiple reflection phenomena. This study provides insights for the design of advanced electronic packaging materials, although further reliability and durability tests are needed to validate practical applicability.

## 1. Introduction

As electronic devices rapidly advance toward higher frequencies, increased integration, and greater power density [1,2,3], effective thermal management and electromagnetic compatibility (EMC) have emerged as critical challenges impacting device performance and reliability. The trend toward higher frequencies in information technology has not only exacerbated electromagnetic interference (EMI) but also amplified electromagnetic radiation from advanced technologies such as 5G communication systems [4] and millimeter-wave radar applications [5]. These intensified electromagnetic emissions heighten the risk of inter-circuit crosstalk, potentially leading to signal distortion, elevated bit error rates, and even complete system failure [6,7,8]. Furthermore, the growing integration density of modern electronic devices necessitates efficient thermal management to maintain optimal operation. Inadequate heat dissipation can significantly impair operational speed and measurement accuracy, ultimately causing performance degradation. Studies have shown that a 10 °C increase in component temperature can reduce device reliability by 50% [9]. To address these dual challenges, conventional approaches typically employ separate electromagnetic shielding solutions to achieve optimal EMI shielding effectiveness, combined with additional thermal management components to enhance heat dissipation. However, this multi-component integration often increases system complexity and space requirements, potentially hindering further device miniaturization. Consequently, multifunctional materials are required to simplify structural design in electronic applications. While highly conductive materials are traditionally considered ideal for electromagnetic shielding due to their effectiveness derived from free electron transport within conductive networks and electromagnetic wave interactions as described by the Simon equation [10], their high conductivity may lead to internal short circuits that disrupt normal device operation. This drawback often necessitates additional insulation measures. Therefore, developing materials that simultaneously integrate electromagnetic shielding capability, thermal dissipation properties, and high electrical insulation characteristics becomes crucial for advancing electronic device technology.

Liquid metals (LMs) are a class of metals with melting points below the fusion temperature of aluminum (660.37 °C). Due to their exceptional properties—such as high electrical conductivity, superior thermal conductivity, favorable specific heat capacity, excellent stability, and outstanding fluidity [11,12]—LMs have garnered significant interest in diverse fields, including flexible electronics [13], soft robotics [14], biomedical engineering [15], and photocatalysis [16]. However, LMs also exhibit several inherent drawbacks, such as high surface tension, poor wettability, excessive fluidity, and a tendency for electrical leakage, which can lead to short circuits in electronic devices. Furthermore, their corrosive nature promotes the formation of intermetallic compounds when in contact with most conventional metals [17,18,19]. Mitigating the leakage issues of LMs could substantially broaden their application potential. Owing to its exceptional electrical conductivity (~3 × 10^6^ S·m^−1^) and thermal conductivity (30~35 W·m^−1^·K^−1^) [20], gallium-indium alloy has been widely utilized as a functional filler to enhance both electromagnetic interference shielding effectiveness (EMI SE) and thermal conductivity in composite materials. Yang et al. [21] fabricated flexible composite films with hierarchical gradient structures consisting of cellulose nanofiber/Fe_3_O_4_, cellulose nanofiber/liquid metal, and cellulose nanofiber/graphene nanoplatelets (CNF/Fe_3_O_4_&CNF/LM&CNF&GNPs) through vacuum filtration and cold-pressing techniques. In a separate approach, Qiu et al. [22] prepared composite materials by selectively distributing liquid metal (LM) and multi-walled carbon nanotubes (CNTs) on thermoplastic polyurethane (TPU) particles using blending and hot-pressing methods. Their method effectively solves the problem of liquid metal leakage, but it is worth noting that the electrical insulation performance of the material is a key requirement for the practical application of electronic packaging systems. The very property that makes LMs attractive, their high conductivity, becomes a significant limitation in electronic applications due to potential leakage that could cause short circuits in circuit boards. To overcome this challenge, researchers have developed microencapsulation techniques to create core-shell structured LM microcapsules that effectively prevent leakage. White [23] successfully encapsulated dicyclopentadiene within urea-formaldehyde microcapsules, creating a material capable of autonomous damage repair. With advancements in microencapsulation technology, LM microencapsulation has expanded the application scope of LMs. Wang et al. [24] fabricated a microencapsulated phase change material (MEPCM) using liquid-phase microencapsulation, comprising a Sn-Bi-In liquid metal (LM) core surrounded by a PMMA shell with internal voids. The researchers prepared composite materials by incorporating the MEPCM into thermogel matrices. At 50 wt% loading, the composite showed an enthalpy of 126.72 J·cm^−3^ and thermal conductivity of 3.23 W·m^−1^·K^−1^, indicating potential applicability for electronic thermal management systems. When liquid metal is microencapsulated, the resulting composite materials exhibit significantly reduced overall electrical conductivity. Since conventional electromagnetic interference (EMI) shielding mechanisms primarily rely on high conductivity, this property alteration renders existing EMI shielding theories inadequate for such insulating composites. Furthermore, microencapsulation also impacts thermal dissipation performance. Therefore, it becomes crucial to develop multifunctional composite materials while elucidating their underlying structure-property relationships.

This study capitalizes on the superior properties of liquid metals while overcoming their leakage limitations through the development of poly(urea-formaldehyde)-encapsulated liquid metal (PUF-LM) microcapsules with well-defined core-shell architectures. Employing a eutectic Gallium-indium alloy as the core material, we fabricated PUF-LM microcapsules via in-situ polymerization. Comprehensive characterization of the microcapsules’ morphology and elemental composition was conducted using scanning electron microscopy (SEM) and X-ray diffraction (XRD) analyses. Through precise optimization of ultrasonic power and stirring rate parameters during synthesis, we successfully obtained four PUF-LM microcapsule variants with controlled particle size distributions. The PUF-LM microcapsules were subsequently incorporated into polymer matrices using a combined blending and freeze-drying approach. We systematically examined the influence of microcapsule size and filler loading on the composites’ thermal conductivity and electrical insulation performance. Moreover, the electromagnetic interference shielding effectiveness (EMI SE) characteristics of these insulating PUF-LM composites were quantitatively analyzed through a microcapacitor structural model, which accounted for variations in both particle size and filler concentration. Our findings provide fundamental insights for designing advanced functional materials, establishing that PUF-LM microcapsule composites represent a promising candidate system for next-generation electronic packaging applications.

## 2. Results

### 2.1. Particle Size Statistics of PUF-LM Microcapsules

**Figure 1:**
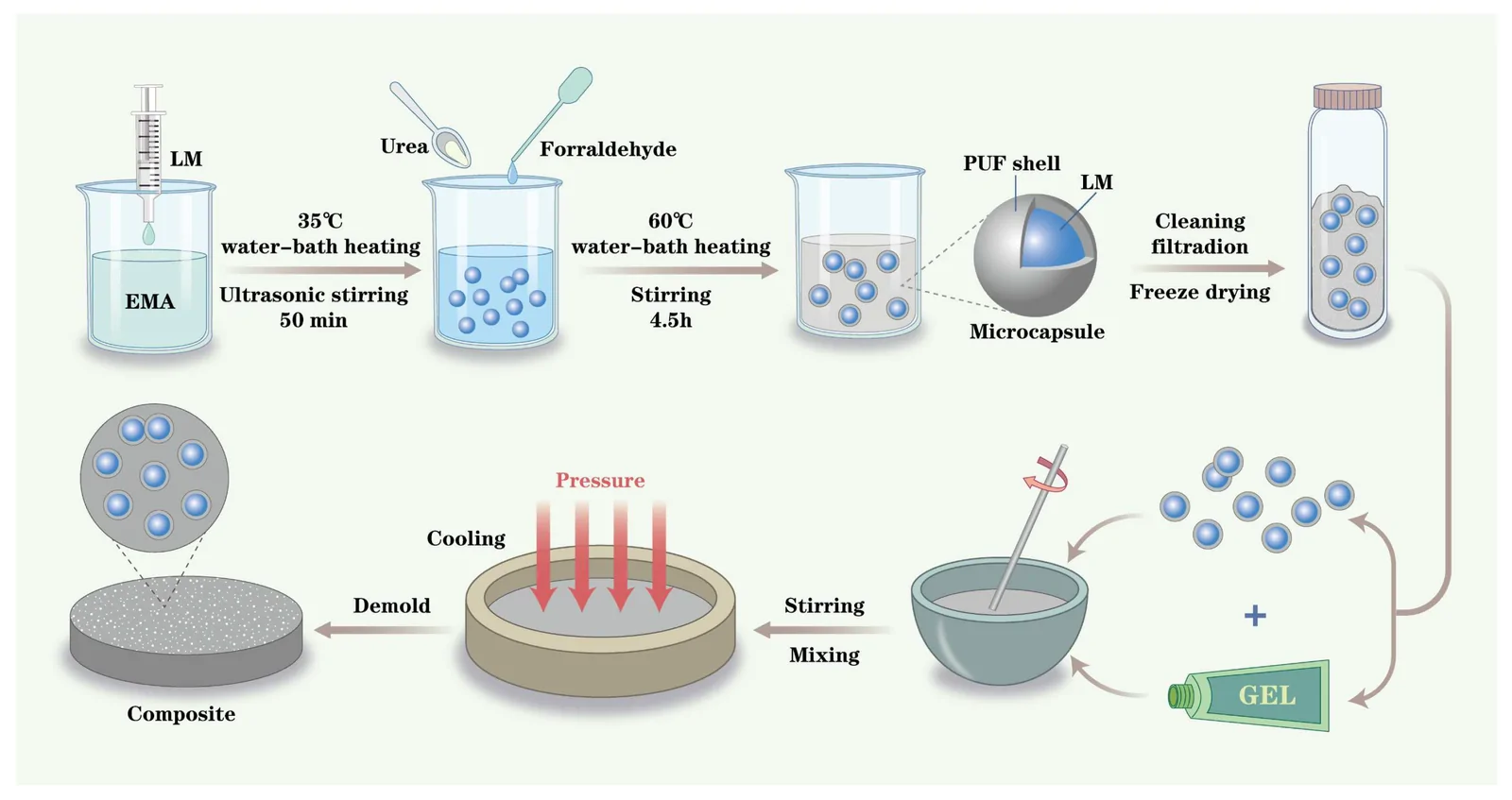
Schematic of the preparation of PUF-LM microcapsules and their composites.

Figure 1 illustrates the schematic preparation process of PUF-LM microcapsules and their composite fabrication. The procedure consists of two main stages. First, PUF-LM microcapsules are synthesized via an in situ polymerization method. Aqueous EMA solution is mixed with deionized water, followed by the addition of resorcinol, NaCl, and urea. The pH is adjusted to 3.5, and the mixture is heated to 35 °C. gallium-indium alloy (LM) is then slowly introduced under ultrasonic irradiation and magnetic stirring. Subsequently, formaldehyde is added, and the reaction continues at 60 °C for 4.5 h to form PUF-encapsulated LM microcapsules, which are then purified by repeated washing and freeze-dried to obtain dry powders. Second, the resulting PUF-LM microcapsule powder is dispersed in a gel matrix through mixing and stirring to fabricate the final composite materials.

**Figure 2:**
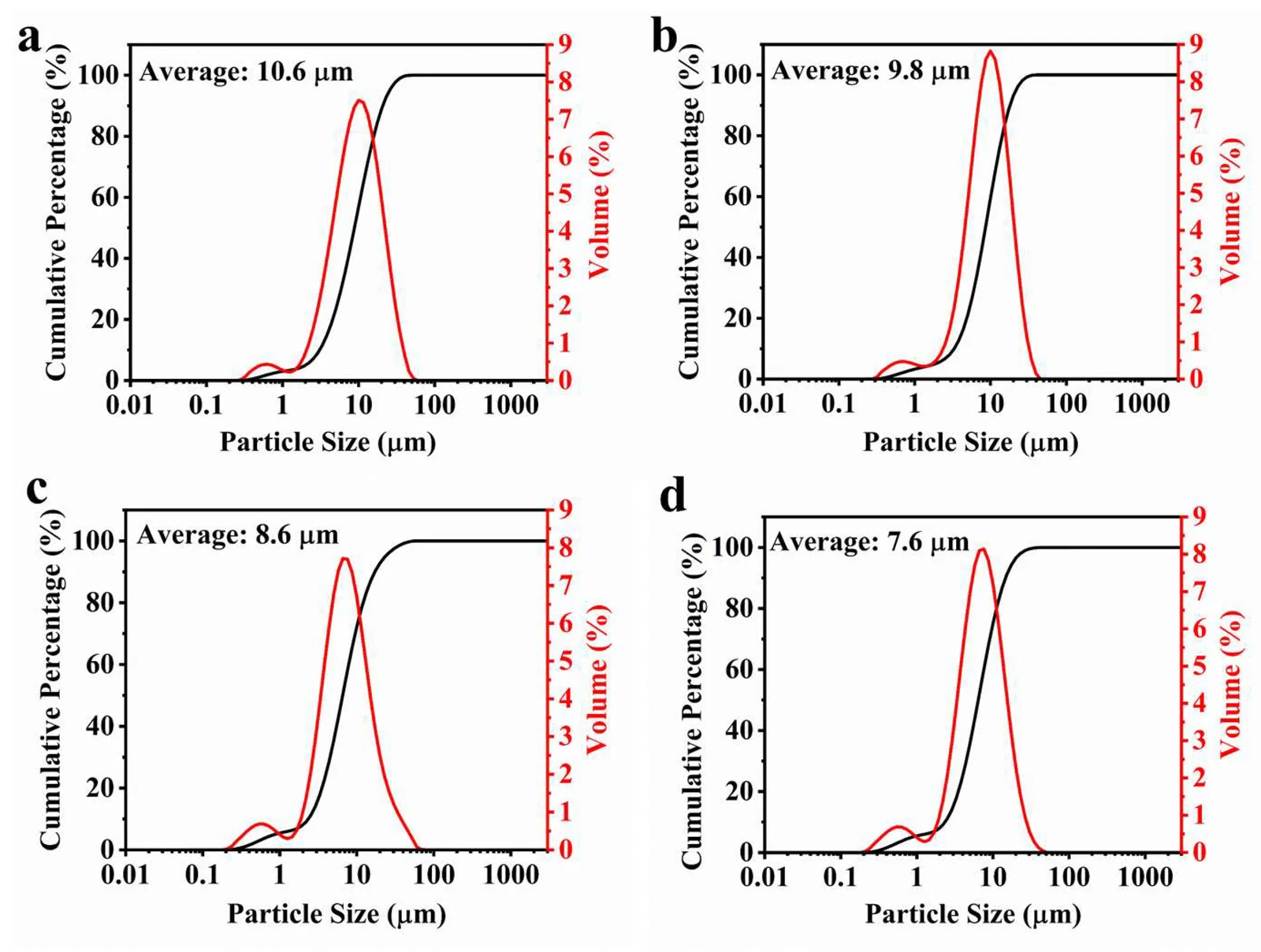
Statistical graph of particle size under different reaction conditions: (**a**) 320 W @ 1200 rpm, (**b**) 400 W @ 1200 rpm, (**c**) 480 W @ 800 rpm, (**d**) 480 W @ 1600 rpm.

The particle size of PUF-LM microcapsules was effectively modulated by varying the stirring rate and ultrasonic power during synthesis. Laser diffraction particle size analysis (Mastersizer 3000, Malvern Panalytical Ltd., Worcestershire, UK) was employed to characterize the size distribution of microcapsules prepared under different processing conditions. Figure 2 presents the statistical analysis of particle size distributions obtained at varying stirring rates and ultrasonic power levels. All four sample groups exhibited volume distribution curves approximating normal distributions, indicating relatively uniform microcapsule sizes were achieved across all preparation conditions.

Figure 2a and Figure 2b display the size distributions for conditions 320 W @ 1200 rpm and 400 W @ 1200 rpm, with mean particle diameters of 10.6 μm and 9.8 μm, respectively. At a constant stirring rate, increased ultrasonic power resulted in smaller microcapsules, attributable to enhanced dispersion and fragmentation of the liquid metal under higher ultrasonic energy input. Figure 2c and Figure 2d present the size distributions for 480 W @ 800 rpm and 480 W @ 1600 rpm conditions, yielding mean diameters of 8.6 μm and 7.6 μm, respectively. When maintaining constant ultrasonic power, higher stirring rates produced smaller microcapsules due to increased shear forces that more effectively broke the liquid metal into finer droplets. These results demonstrate that precise control over microcapsule size can be achieved through systematic adjustment of ultrasonic power and stirring rate parameters during synthesis. The established size-control methodology provides a foundation for subsequent investigations into size-dependent properties of the microcapsules.

### 2.2. Morphological and Structural Characterization of PUF-LM Microcapsules

**Figure 3:**
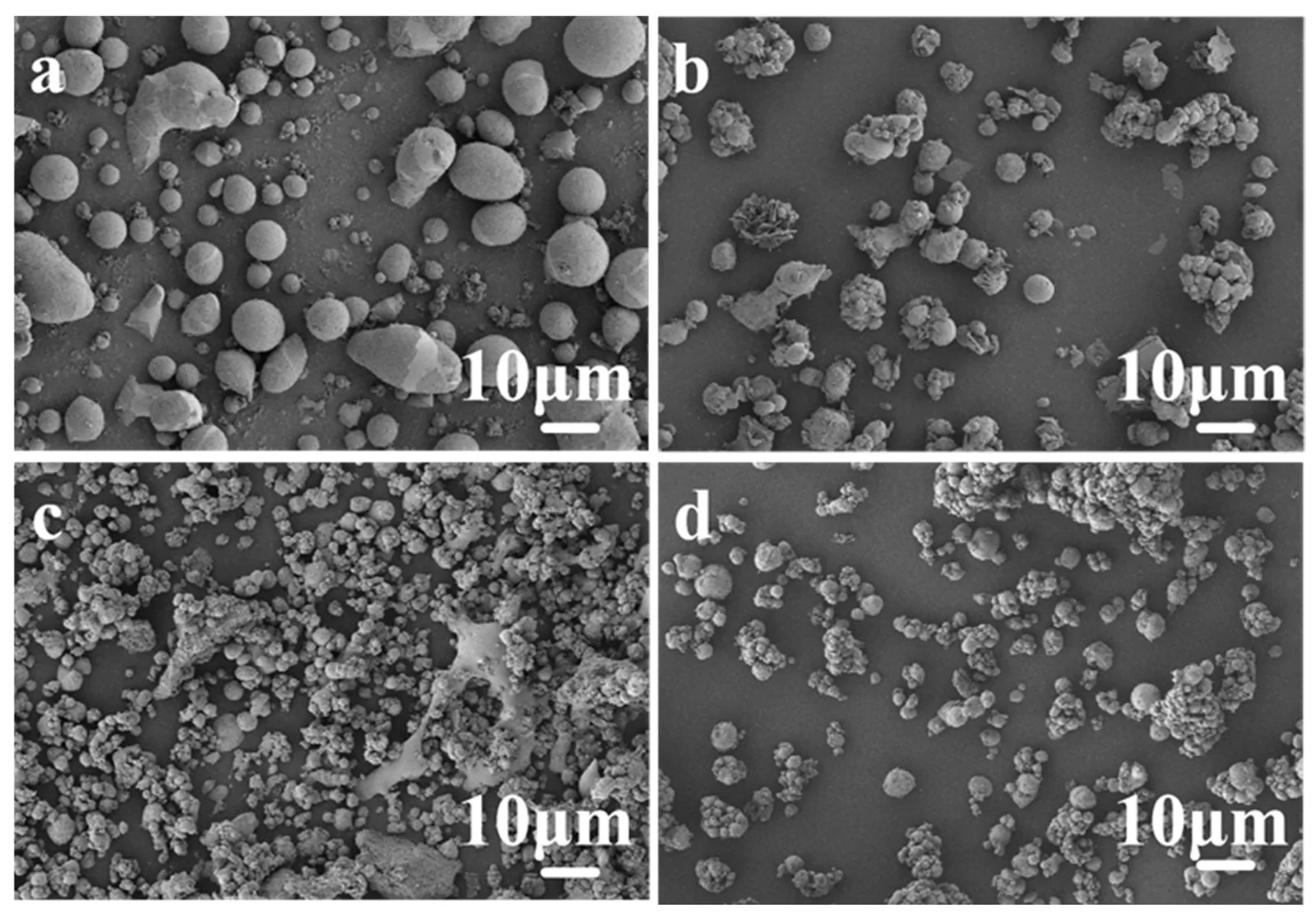
SEM for different particle sizes (**a**) 10.6 μm, (**b**) 9.8 μm, (**c**) 8.6 μm, (**d**) 7.6 μm.

The PUF-LM microcapsules were fabricated via in situ polymerization, forming a poly(urea-formaldehyde) (PUF) shell layer on the surface of gallium-indium alloy droplets. Figure 3 presents SEM images of PUF-LM microcapsules with four different average particle sizes, arranged in descending order. As shown in Figure 3 and Figure S1 (Supplementary Materials), all microcapsules exhibit a spherical morphology. Numerous closely packed submicron-sized particles are clearly visible on the microcapsule surface. These particles are PUF nanoparticles formed from linear PUF prepolymers, which are produced by the polymerization of methylol urea and amino groups under alkaline conditions at elevated temperatures. Regardless of particle size, all PUF-LM microcapsules display relatively intact surfaces without obvious rupture or defects.

As illustrated in Figure 3a,b, the 10.6 μm and 9.8 μm microcapsules are well dispersed. In contrast, the 8.6 μm and 7.6 μm microcapsules (Figure 3c,d) exhibit some degree of agglomeration. This agglomeration is attributed to the high surface tension of the liquid metal itself. As the droplet size decreases, the interparticle distance becomes smaller, and van der Waals forces become more pronounced, thereby promoting agglomeration [25]. Additionally, when the liquid metal is dispersed into finer droplets, the surface charge distribution may become uneven, leading to increased local electrostatic attraction and further contributing to microcapsule agglomeration [26]. Overall, the SEM characterization confirms that PUF-LM microcapsules with various particle sizes and good spherical morphology were successfully prepared.

**Figure 4:**
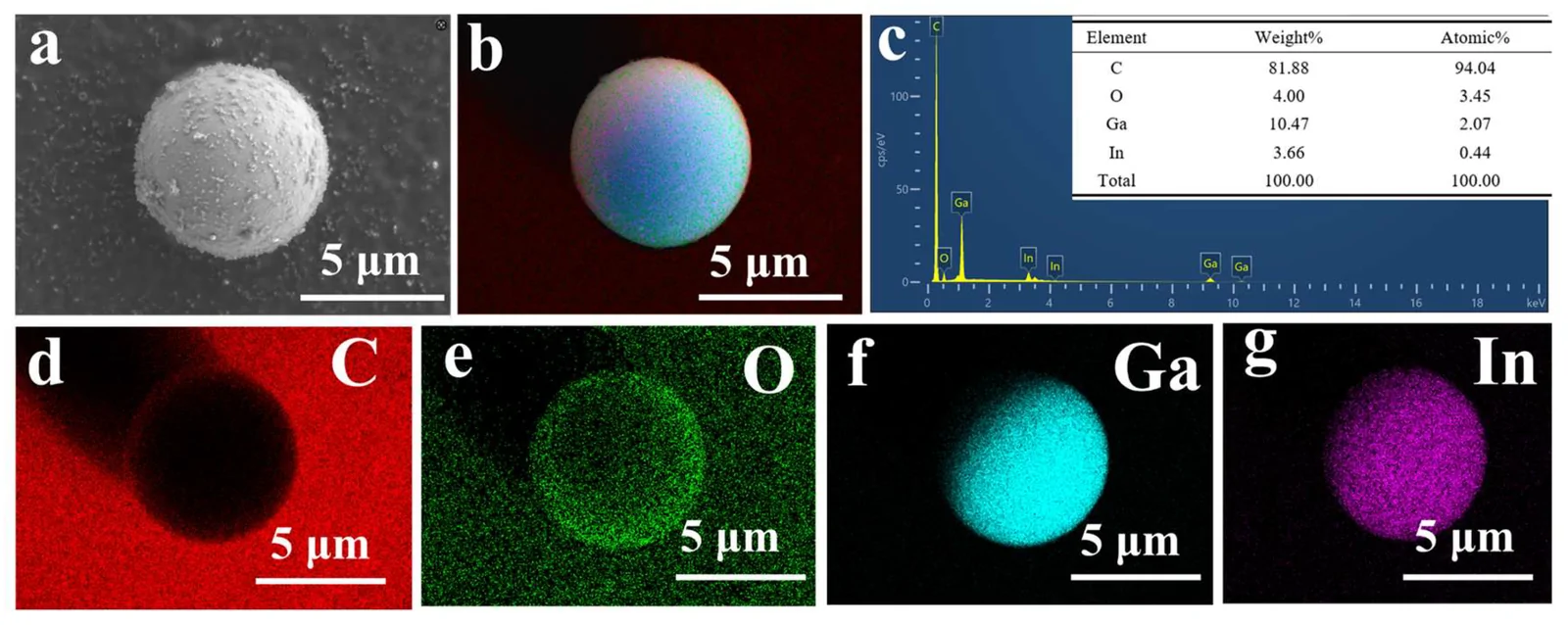
(**a**) SEM image of 10.6 μm microcapsule, (**b**) layered image, (**c**) total elemental spectrum of the microcapsule, (**d**) C, (**e**) O, (**f**) Ga, (**g**) In.

Figure 4 presents the EDS analysis of the 10.6 μm PUF-LM microcapsules, revealing their elemental composition and distribution. As shown in Figure 4c, the microcapsules consist primarily of four elements: C, O, Ga, and In, with atomic percentages of 94.04%, 3.45%, 2.07%, and 0.44%, respectively. Based on the preparation process, it can be inferred that the shell layer is composed of the organic polymer PUF, while the Ga and In elements originate from the liquid metal core. The C and O elements are mainly contributed by the PUF shell. The elemental mappings of C, O, Ga, and In (Figure 4d–g) are consistent with the microcapsule morphology shown in Figure 4a,b. The EDS spectra of the 9.8 μm, 8.6 μm, and 7.6 μm microcapsules are provided in Figure S2 of the Supplementary Materials.

**Figure 5:**
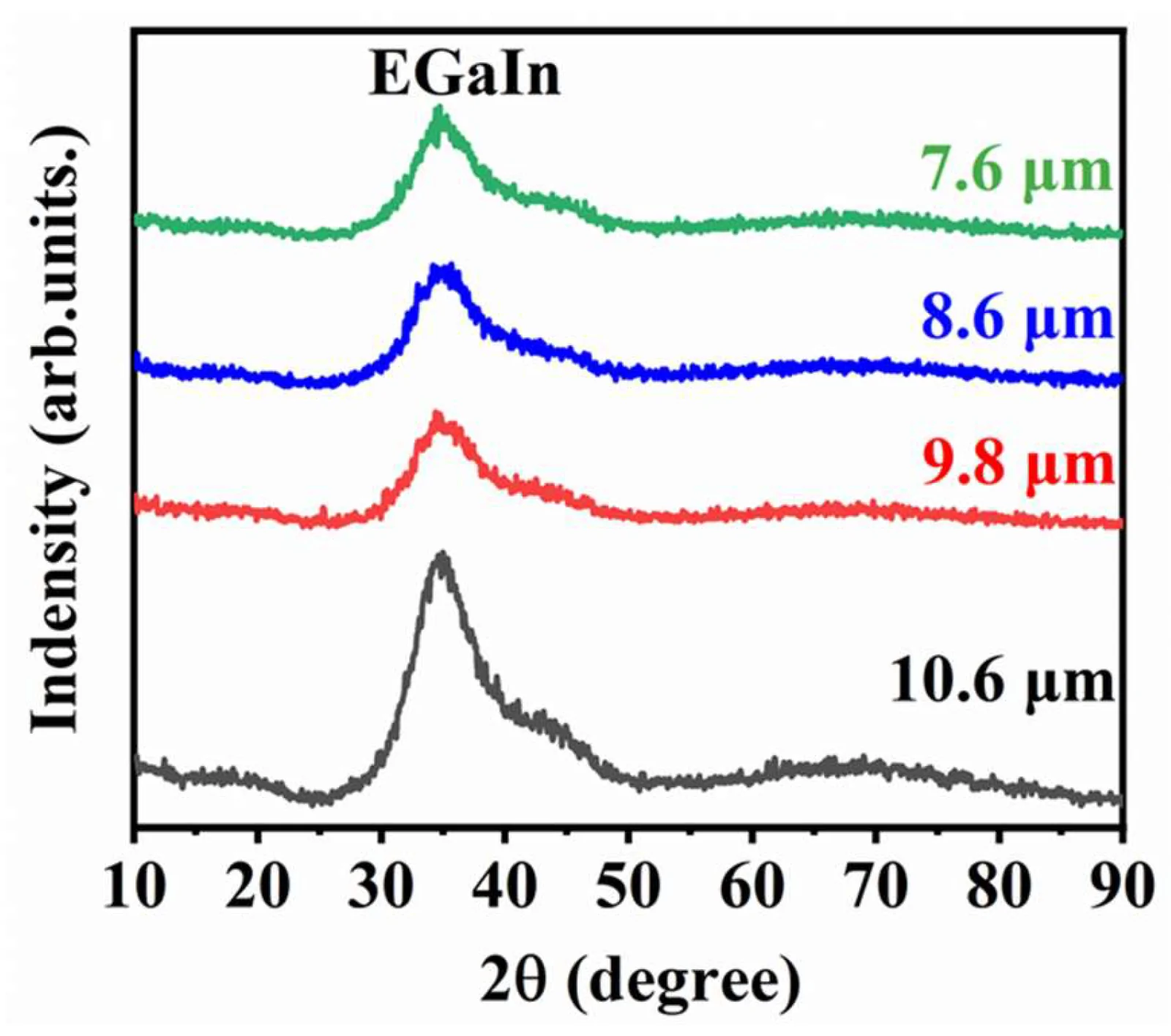
XRD plots of PUF-LM microcapsules with different particle sizes.

Figure 5 shows the XRD patterns of the PUF-LM microcapsules with four different particle sizes. A prominent diffraction peak is observed at approximately 35°, which, upon comparison with the standard PDF card, corresponds to the crystal structure of the gallium-indium alloy. No obvious oxidation peaks are detected, indicating a low degree of surface oxidation. The SEM morphology, EDS, and XRD analysis collectively demonstrate that the PUF shell effectively encapsulates the liquid metal core. By varying the ultrasonic power and stirring rate during the reaction, PUF-LM microcapsules with different particle sizes were successfully prepared in this study.

It should be noted that the four microcapsule populations were synthesized under different ultrasonic powers and stirring rates, which primarily affect the emulsification of the liquid metal into droplets of varying diameters. The subsequent in situ polymerization of the PUF shell was performed under identical chemical conditions, including the same monomer concentrations, pH, temperature, and reaction time, for all batches. Furthermore, the XRD patterns confirm that the Ga-In alloy cores are equally well encapsulated and exhibit negligible oxidation in all cases. Therefore, while minor variations in shell thickness or core-to-shell ratio cannot be entirely excluded, they are considered secondary to the dominant effect of microcapsule diameter. The property trends reported in the following sections are interpreted primarily in terms of particle size, which is the key design parameter deliberately controlled in this study.

### 2.3. Thermal Performance of LMMCs

**Figure 6:**
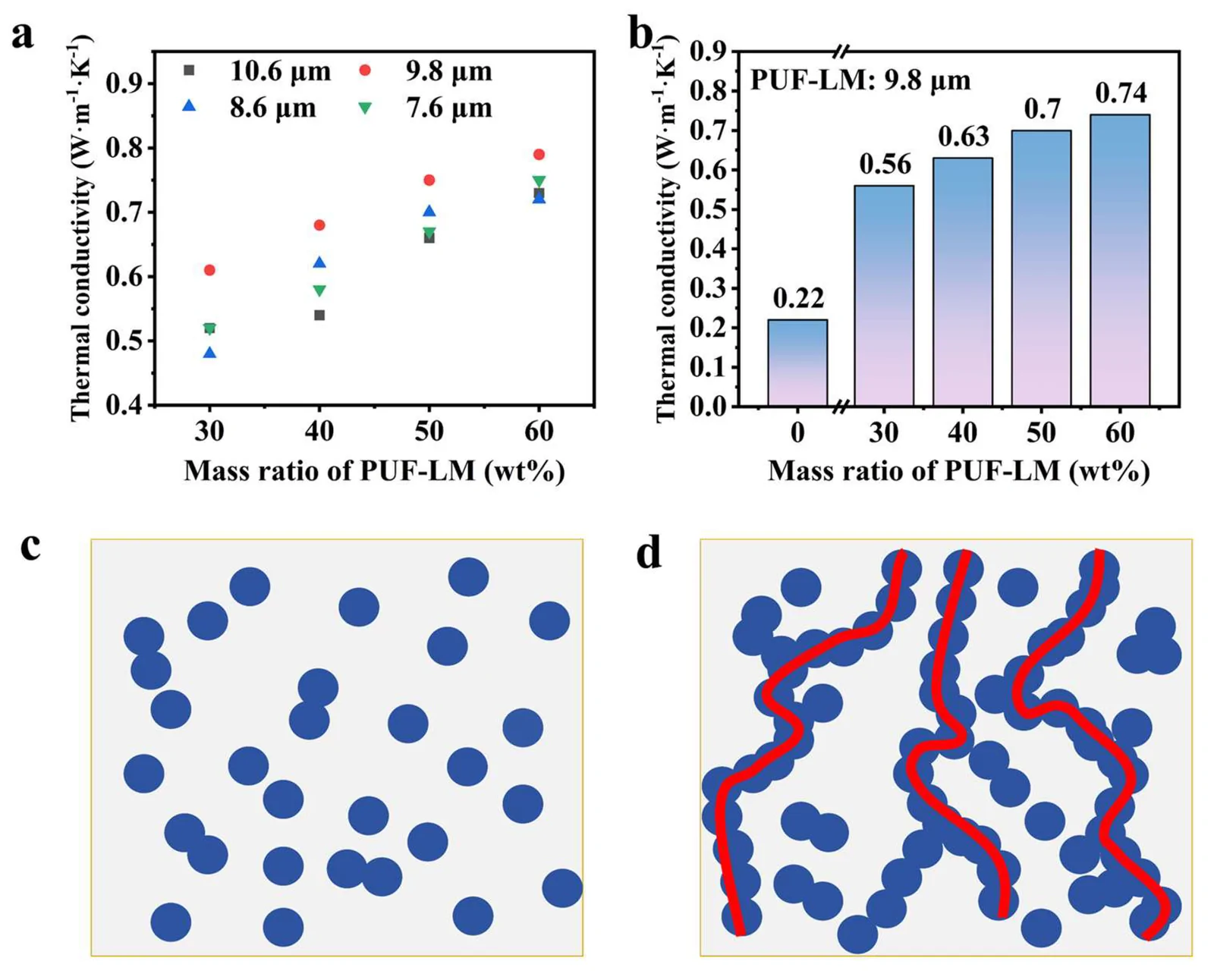
(**a**) Variation in thermal conductivity of PUF-LM microcapsule composites with different particle sizes and contents of microcapsules, (**b**) variation in thermal conductivity of PUF-LM microcapsule composites with different contents of 9.8 μm microcapsules, (**c**) island structure with low filler, (**d**) thermal conductivity paths with enough filler.

In solid polymers, heat transfer is primarily governed by phonon transport, with thermal conduction occurring mainly through lattice vibrations and free electron motion [27,28]. In this study, four types of PUF-LM microcapsules with different particle sizes were prepared to investigate the size-dependent effects on the thermal conductivity of the resulting composites. The thermal conductivity of LMMCs containing these four microcapsule types at various loading fractions (30 wt%, 40 wt%, 50 wt%, and 60 wt%) was measured using a thermal conductivity analyzer, and the results are presented in Figure 6. Figure 6a shows the thermal conductivity variation in LMMCs as a function of microcapsule size and loading content. As the particle size decreases, the thermal conductivity first increases and then decreases. Figure 6b displays the thermal conductivity of LMMCs containing 9.8 μm microcapsules at different loading fractions. The maximum thermal conductivity reaches 0.53 W·m^−1^·K^−1^ at a loading of 60 wt% for the 9.8 μm microcapsules. Compared to the pure silicone matrix (0.22 W·m^−1^·K^−1^) without any filler, this represents a 139.6% enhancement, demonstrating a notable enhancement in the thermal conductive performance of LMMCs under the tested conditions.

**Figure 7:**
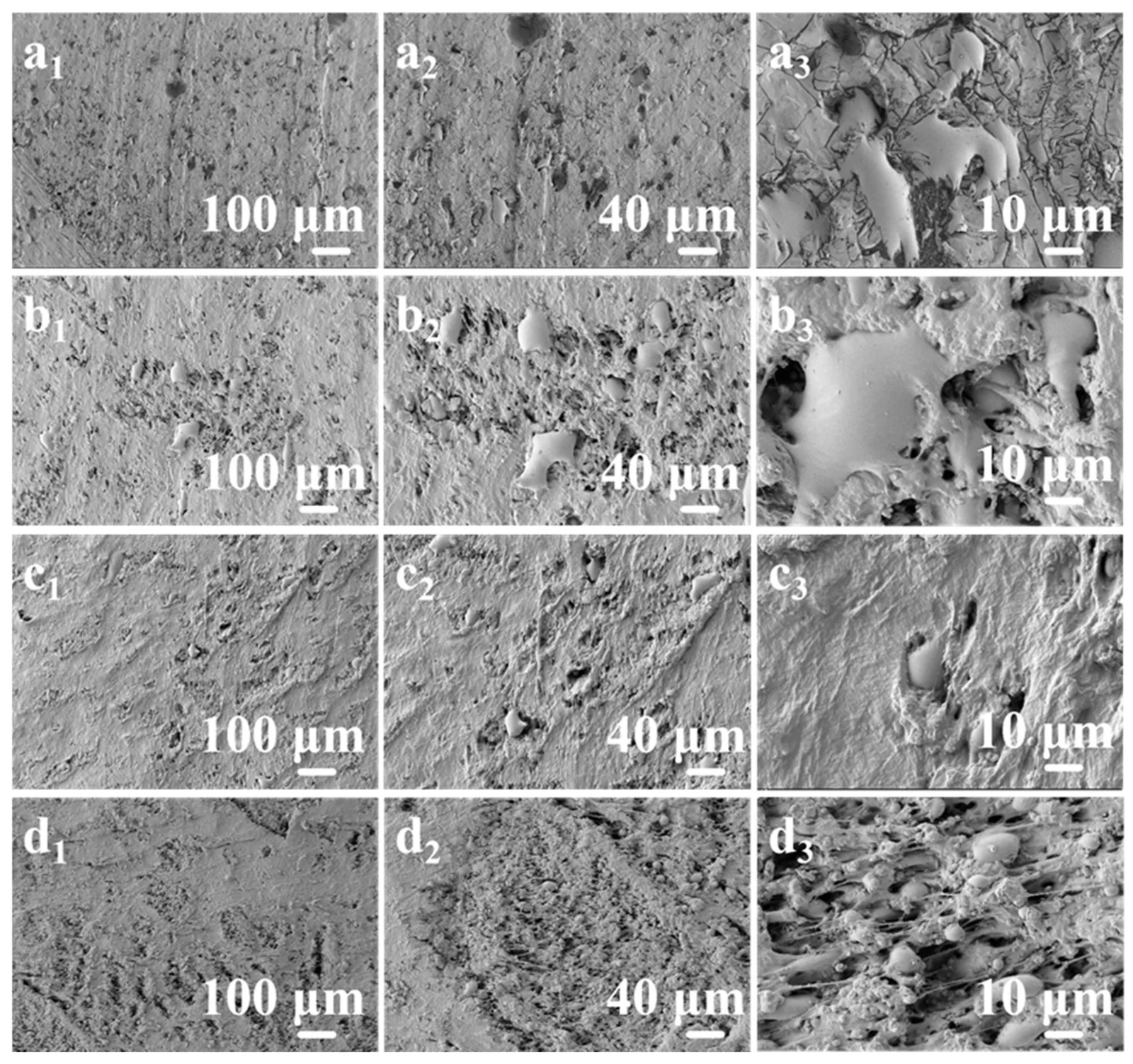
SEM cross-sectional images of PUF-LM composite materials with 60 wt% microcapsules of different particle sizes added (**a1**–**a3**)10.6 μm, (**b1**–**b3**) 9.8 μm, (**c1**–**c3**) 8.6 μm, (**d1**–**d3**) 7.6 μm.

To investigate the thermal conduction mechanism within the PUF-LM composites, cross-sectional SEM analysis was performed on LMMCs containing different microcapsule sizes (10.6 μm, 9.8 μm, 8.6 μm, and 7.6 μm) at a constant loading of 60 wt%, as shown in Figure 7. Figure 7(a1–a3) present the microstructural SEM images of the 10.6 μm sample, Figure 7(b1–b3) for 9.8 μm, Figure 7(c1–c3) for 8.6 μm, and Figure 7(d1–d3) for 7.6 μm. Simulation results in Supplementary Figure S3 demonstrate that smaller microcapsules result in a higher particle density within the matrix at equivalent mass fractions. Figure 7 reveals that larger microcapsules (10.6 μm and 9.8 μm) exhibit liquid metal rupture in their cross-sections during sample preparation, while smaller microcapsules (8.6 μm and 7.6 μm) maintain structural integrity with more compact packing. This size-dependent mechanical behavior indicates that larger microcapsules are more prone to fracture during sectioning due to their reduced structural stability under shear stress.

Analysis of the cross-sectional SEM images reveals that as the microcapsule size decreases, the interparticle gaps narrow and the PUF-LM microcapsules become more densely packed. This leads to an increased thermal network density and more complete heat conduction pathways [29,30], which initially enhances thermal conductivity. However, when the microcapsule size decreases beyond a critical point, the thermal conductivity exhibits a declining trend. This phenomenon occurs because excessive particle proximity restricts phonon transport. According to the Debye equation, when the specific heat capacity and phonon velocity remain constant, a reduced interparticle distance shortens the phonon mean free path [31,32,33], consequently decreasing the overall thermal conductivity. Therefore, the thermal conductivity of LMMCs first increases and then decreases with reducing particle size, reaching its maximum value at 9.8 μm, where optimal microcapsule packing and phonon transport conditions are achieved.

It should be noted that the observed thermal conductivity maximum at 9.8 μm is not solely determined by packing density but also reflects the trade-off among shell-related interfacial resistance, agglomeration-induced voids, and capsule rupture effects. Several factors contribute to this non-monotonic size dependence. First, although the PUF shell thickness is similar for all microcapsule sizes, the shell occupies a larger volume fraction in smaller microcapsules due to their higher specific surface area. Since the thermal conductivity of the PUF shell is much lower than that of the Ga-In alloy core, a higher shell fraction increases the interfacial thermal resistance and reduces the effective thermal conductivity of each microcapsule. Second, agglomeration of smaller microcapsules (observed in Figure 3c,d) introduces additional voids and reduces the contact area between neighboring microcapsules, further impeding heat transfer. Third, cross-sectional SEM images (Figure 7) show that larger microcapsules (10.6 μm and 9.8 μm) are more susceptible to rupture during processing. Although ruptured capsules may release liquid metal, the exposed alloy rapidly forms an insulating oxide layer, which does not create conductive pathways but may introduce local thermal discontinuities. The interplay among these factors, including packing density, shell volume fraction, agglomeration, and rupture, results in the optimal thermal conductivity at 9.8 μm, where a balance between dense packing and minimal adverse effects is achieved.

**Figure 8:**
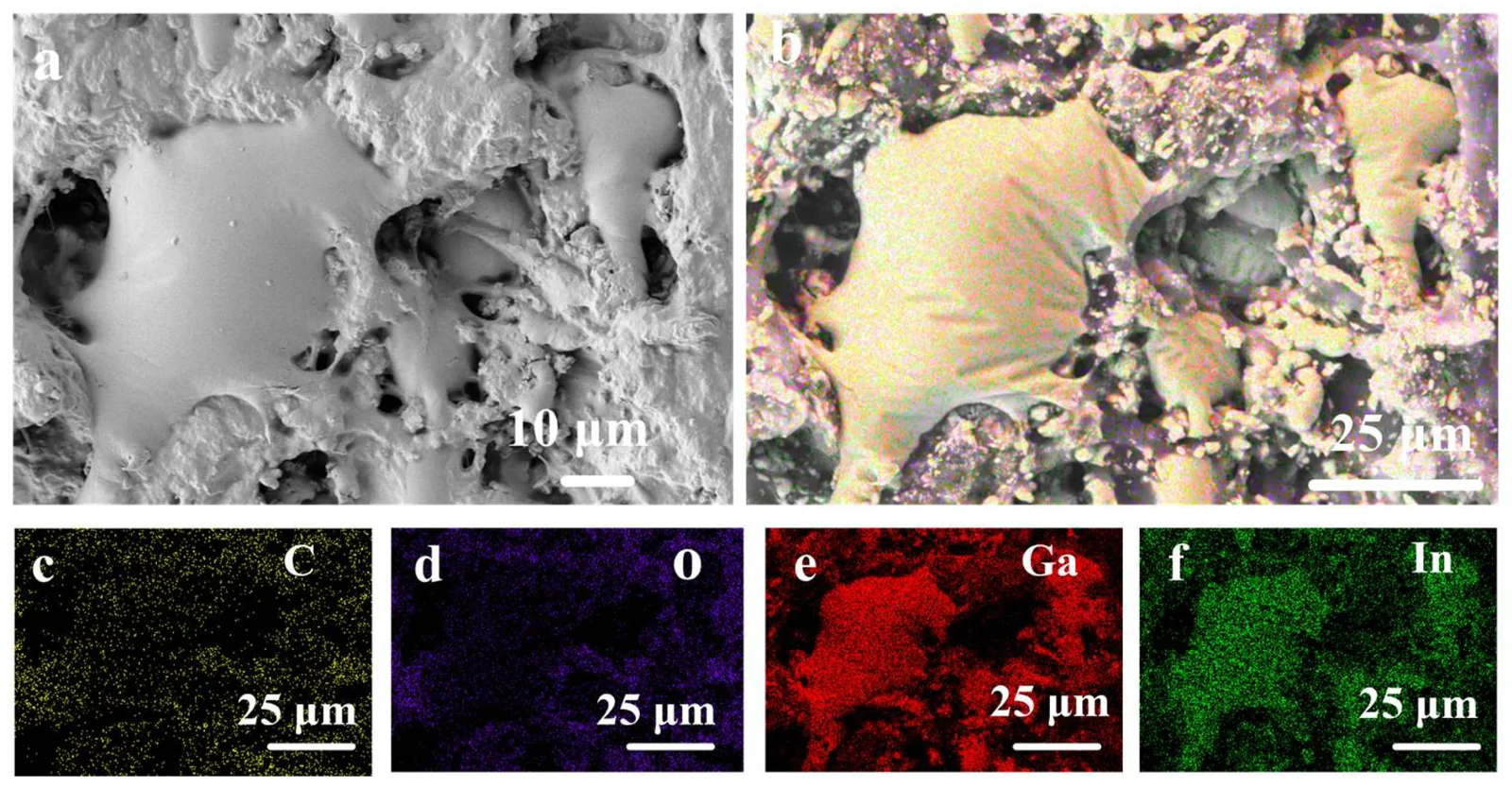
(**a**) Cross-sectional SEM image of a 9.8 μm PUF-LM composite with 60 wt% microcapsule, (**b**) merged EDS elemental mapping showing the distribution of C, O, Ga, and In, (**c**) C elemental mapping, (**d**) O elemental mapping, (**e**) Ga elemental mapping, (**f**) In elemental mapping.

The cross-sections of LMMCs containing 9.8 μm microcapsules at different loading fractions (30 wt%, 40 wt%, 50 wt%, and 60 wt%) were examined by SEM, as shown in Supplementary Materials Figure S5 (a1–a3: 30 wt%, b1–b3: 40 wt%, c1–c3: 50 wt%). Figure 6b demonstrates that for 9.8 μm microcapsules, the thermal conductivity increases with filler content until reaching a critical concentration. At lower loadings (Figure 6c schematic), the microcapsules form isolated island structures [34] within the matrix without establishing complete thermal conduction pathways, resulting in poorer thermal performance. When the filler content exceeds the percolation threshold (Figure 6d schematic), the microcapsules form interconnected networks [35] that significantly enhance heat transfer, achieving maximum thermal conductivity. EDS elemental mapping (Figure 8) of the 60 wt% 9.8 μm sample confirms the uniform distribution of microcapsules in the silicone matrix, with Figure 8c–f showing homogeneous dispersion of C, O, Ga, and In elements. Additional EDS spectra for other particle sizes and loading ratios are provided in Figure S5.

### 2.4. Insulating Properties of LMMCs

**Figure 9:**
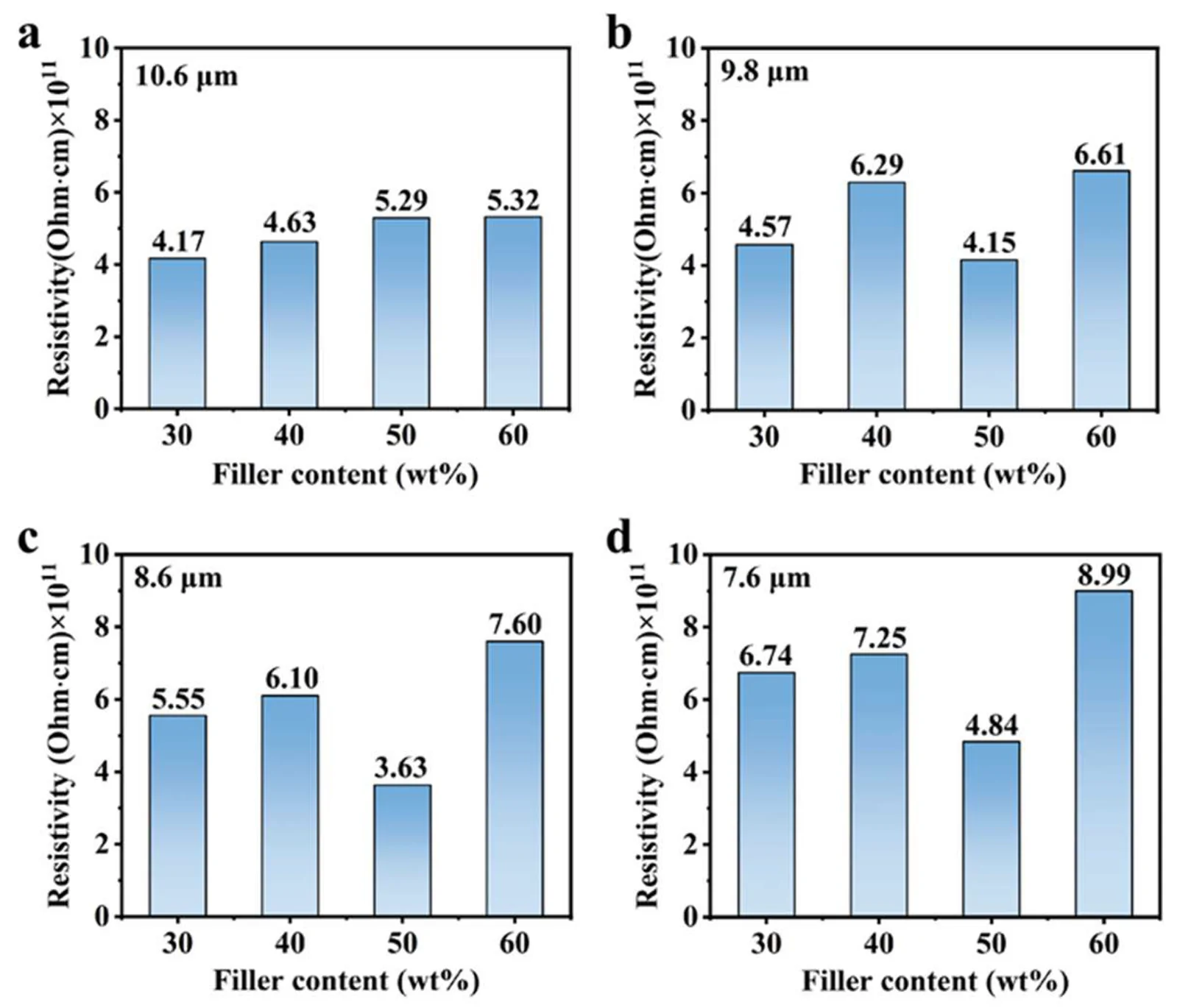
Resistivity variation in PUF-LM composites with different addition content of microcapsules with different particle sizes (**a**) 10.6 μm, (**b**) 9.8 μm, (**c**) 8.6 μm, (**d**) 7.6 μm.

In electronic packaging applications, the resistivity requirements for composite materials depend on the specific application scenario and functional demands [36]. As packaging materials, composites must exhibit excellent electrical insulation properties, typically with a resistivity exceeding 10^9^ Ω·cm, to prevent current leakage and short circuits [37]. Figure 9 presents the resistivity measurements of LMMCs containing PUF-LM microcapsules of different sizes (10.6 μm, 9.8 μm, 8.6 μm, and 7.6 μm) at loading fractions ranging from 30 wt% to 60 wt%. The results demonstrate that the resistivity increases with decreasing particle size. This trend can be attributed to several factors observed in SEM analysis. First, smaller microcapsules possess a higher specific surface area, which increases the interfacial contact resistance between the filler and the matrix [38]. Second, the agglomeration tendency of smaller particles, as evidenced by SEM observations, impairs their dispersion and hinders the formation of conductive networks [39]. Third, enhanced interface effects and a higher density of defects at reduced particle sizes impede electron transport [40]. The maximum resistivity of 8.99 × 10^11^ Ω·cm was achieved with 7.6 μm particles at a loading of 60 wt%.

For composites containing 10.6 μm microcapsules (Figure 9a), the resistivity gradually increased with filler content without reaching a percolation threshold, which is consistent with percolation theory [41]. In addition to the classical percolation mechanism, charge trapping at the filler-matrix interface plays an important role in determining the electrical resistivity. The PUF shell of the microcapsules and the silicone matrix form a large number of interfaces. Smaller microcapsules possess a higher specific surface area, thereby providing a greater density of interfacial trap states. These traps capture mobile charge carriers and hinder their transport, effectively increasing the resistivity. This effect is superimposed on the percolation behavior. At low filler loadings, the disruption of existing conductive paths dominates. Near the percolation threshold, the formation of tenuous conductive networks competes with charge trapping. At high loadings, agglomeration and increased interfacial resistance further elevate the resistivity. The combination of these mechanisms explains the complex resistivity trends observed in Figure 9, particularly the higher resistivity values achieved with smaller microcapsules. In contrast, composites containing 9.8 μm, 8.6 μm, and 7.6 μm particles (Figure 9b–d) exhibited an initial increase in resistivity, followed by a decrease and a subsequent rise. This behavior is influenced by conductive pathway formation [42], filler dispersion [43], and interface effects [44]. The initial increase occurs because a low filler content disrupts the existing conductive paths within the matrix [45,46]. The subsequent decrease reflects the attainment of the percolation threshold, where electron transport begins to occur through direct filler contacts [47,48]. The final rise in resistivity results from filler agglomeration and increased interfacial resistance at excessive loadings. SEM analysis confirmed that the 10.6 μm particles exhibited better dispersion, which explains why they did not reach the percolation threshold. In summary, three main conclusions can be drawn from this study. First, smaller particles yield higher resistivity at equivalent loading fractions. Second, smaller particles more readily achieve the percolation threshold. Third, all tested composites maintained sufficient insulation for electronic packaging requirements, with a minimum resistivity of 3.63 × 10^11^ Ω·cm.

### 2.5. Electromagnetic Shielding Effectiveness of LMMCs

The electromagnetic interference (EMI) shielding effectiveness (SE) of the LMMCs was measured in the X-band frequency range (8–12 GHz) using a vector network analyzer. Two sets of experiments were conducted. First, to investigate the effect of microcapsule loading content, composites containing 9.8 μm microcapsules at varying loadings (30 wt%, 40 wt%, 50 wt%, and 60 wt%) were evaluated. Second, to examine the effect of particle size, composites with a fixed loading of 60 wt% were prepared using four different microcapsule sizes (10.6 μm, 9.8 μm, 8.6 μm, and 7.6 μm).

**Figure 10:**
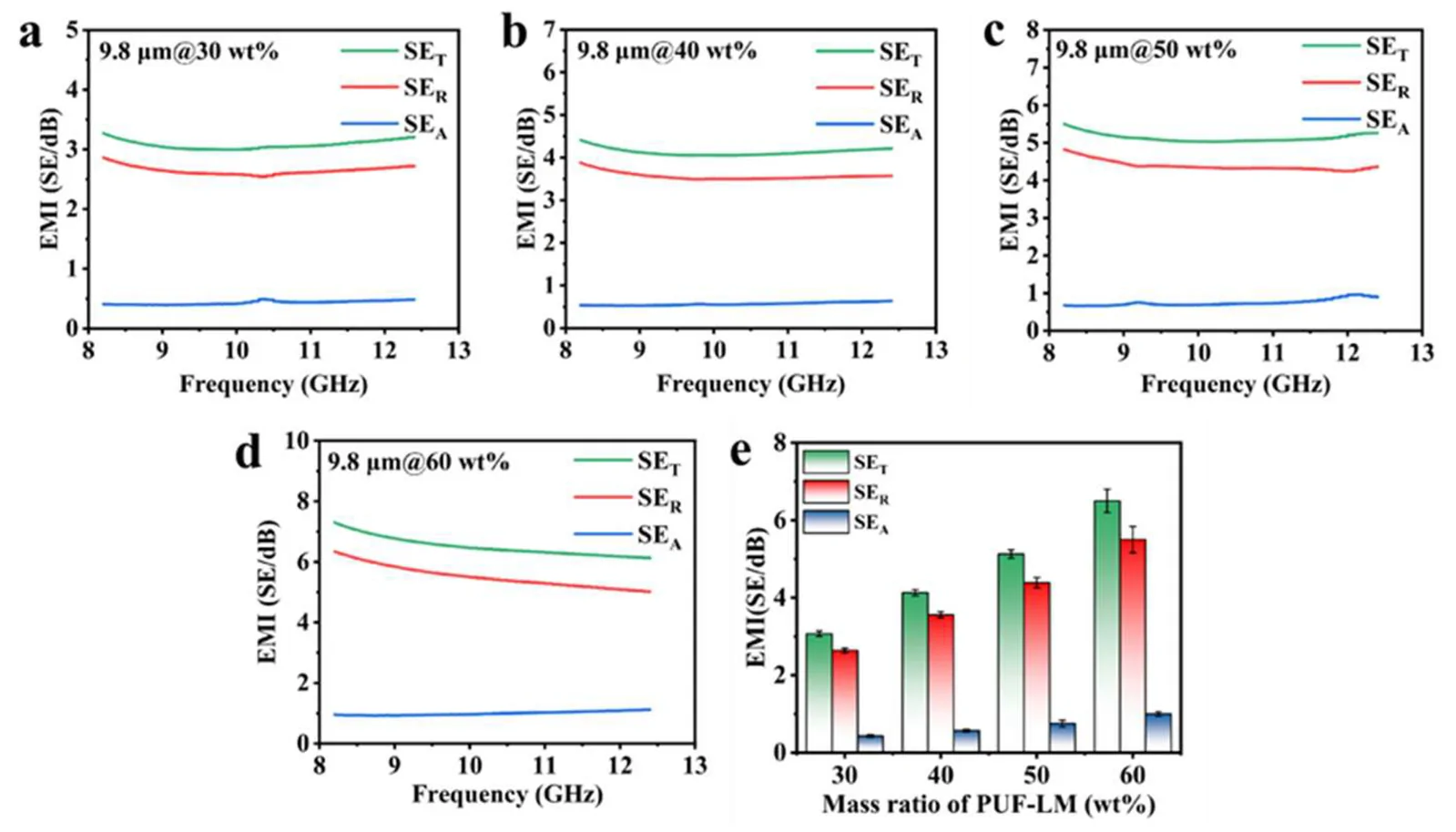
The 9.8 μm particle size microcapsules with different levels of electromagnetic shielding efficacy: (**a**) 30 wt%, (**b**) 40 wt%, (**c**) 50 wt%, (**d**) 60 wt%, (**e**) average electromagnetic shielding efficacy of 9.8 μm PUF-LM composites with different contents.

As shown in Figure 10, when the microcapsule size was maintained at 9.8 μm and the loading content increased from 30 wt% to 60 wt%, the average total SE (SET) values were 3.07 dB, 4.10 dB, 5.16 dB, and 6.50 dB, respectively. This progressive increase demonstrates that higher microcapsule loading enhances EMI shielding performance when the particle size remains constant.

**Figure 11:**
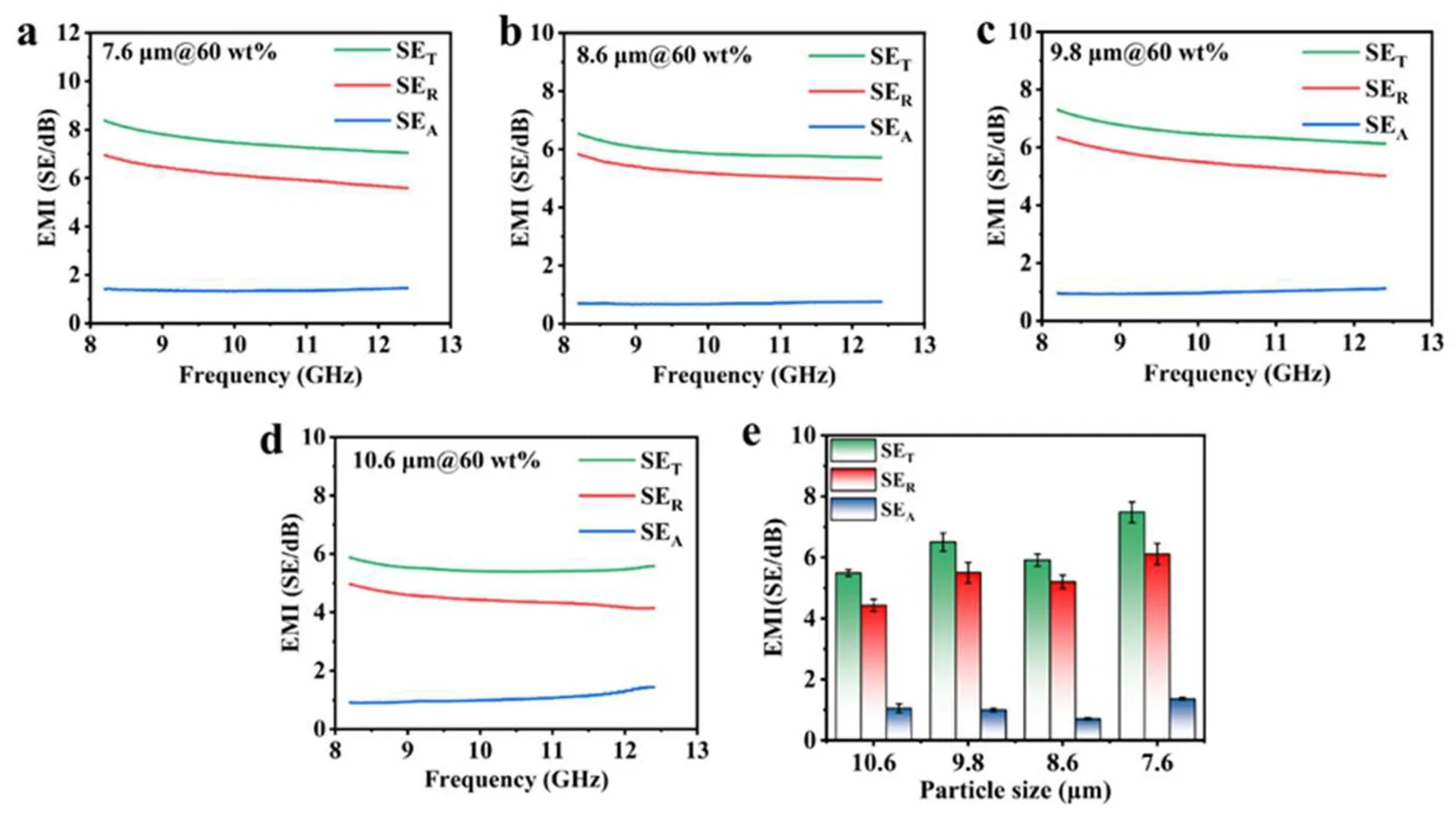
Electromagnetic shielding efficacy of PUF-LM composites with the same content of 60 wt% microcapsules with different particle sizes (**a**) 10.6 μm, (**b**) 9.8 μm, (**c**) 8.6 μm, (**d**) 7.6 μm, (**e**) average electromagnetic shielding efficacy of 60 wt% microcapsules with different particle sizes.

At a fixed loading of 60 wt%, the average SET values for microcapsule sizes of 10.6 μm, 9.8 μm, 8.6 μm, and 7.6 μm were 5.49 dB, 6.50 dB, 5.91 dB, and 7.48 dB, respectively (Figure 11). These results indicate that the EMI SE is influenced by both the particle size and the loading content. The non-monotonic variation in SE with decreasing particle size reflects the competing effects of microcapacitor density, particle agglomeration, and interfacial polarization. Collectively, these findings systematically characterize the composition–structure–property relationships governing the EMI shielding behavior of these insulating composites.

### 2.6. Electromagnetic Shielding Mechanism of Microcapacitor Structure

Compared with conventional electromagnetic shielding materials, LMMCs exhibit several distinct differences, as the shielding effectiveness (*SE*) of materials typically follows theoretical predictions based on the Simon equation [49,50]:

(1) $$SE\left(dB\right)=50+10\log\left(\frac{\sigma }{f}\right)+1.7d\sqrt{\left(\sigma f\right)}$$

In the equation, σ represents the composite’s electrical conductivity (S/cm), f is the electromagnetic wave frequency (MHz), and d is the composite thickness (cm). According to the Simon equation, when the composite thickness and electromagnetic wave frequency remain constant, higher electrical conductivity leads to better shielding effectiveness (*SE*). Theoretically, the shielding capability of conductive materials primarily stems from free electron transport within conductive networks and their interaction with electromagnetic waves [51,52,53]. However, the Simon equation’s applicability decreases when resistivity exceeds 10^3^ Ω·m and becomes completely invalid for insulating materials with resistivity above 10^8^ Ω·m [54]. The LMMCs in this study exhibit resistivity exceeding 10^8^ Ω·m, indicating extremely low conductivity that invalidates the Simon equation, as the microcapsules cannot form conductive networks within the silicone matrix. Consequently, the observed EMI shielding must originate from insulating mechanisms, with SE being dependent on the size and concentration of the incorporated PUF-LM microcapsules. The structural model of the microcapacitor consists of PUF-LM microcapsules with a Ga-In alloy core and a poly(urea-formaldehyde) shell forming a core–shell structure, which are uniformly dispersed in a silicone rubber matrix. This microcapacitor model elucidates the interaction between electromagnetic shielding and the microcapsules, thereby achieving effective EMI shielding performance. EDS mapping confirms the presence of Ga/In elements in the shell with mobile electrons, where adjacent PUF-LM microcapsules act as electrode plates and the intervening silicone matrix serves as a dielectric layer, forming pseudo-parallel-plate microcapacitors. The surface free electrons on microcapsules generate induced charges under incident electromagnetic waves, causing periodic shell oscillations that produce microcurrents, which convert electromagnetic energy into heat through ohmic loss [55,56]:

(2) $$W_{0}=\sum_{i=1}^{n}I^{2}R_{i}$$

In the equation, *W*_0_ represents the total power dissipation due to ohmic losses, while *I* and *R_i_* denote the current intensity and resistance of each microcapacitor electrode plate, respectively. The conductive shell layer on PUF-LM microcapsules generates strong electromagnetic wave reflections, where multiple reflections enhance ohmic losses in the shell layer and absorb a portion of electromagnetic energy, thereby contributing partially to the overall shielding effectiveness (*SE*) through ohmic dissipation mechanisms. For a uniform spherical microcapsule, the resistance can be expressed as

(3) $$R=\rho \cdot \frac{d}{A}$$

In the equation, *ρ* represents the material’s resistivity (Ω·m), *A* denotes the cross-sectional area for current conduction (m^2^), and *d* corresponds to the conduction path length (m). For spherical microcapsules, d can be approximated as the particle diameter. Through derivation from the spherical volume and cross-sectional area relationships with radius r, it can be deduced that

(4) $$R\propto \frac{1}{r}\propto \frac{1}{\sqrt[{3}]{V}}$$

According to Ohm’s law,

(5) $$I\propto \sqrt[{3}]{V}$$

(6) $$W_{0}\propto \sum_{i=1}^{n}\sqrt[{3}]{V_{i}}$$

When considering the microcapsules as electrode plates, the plate resistance is inversely proportional to the cube root of the microcapsule volume, while the current is directly proportional to the cube root of the volume. According to the ohmic loss mechanism, the dissipated electromagnetic wave energy is proportional to the cube root of the microcapsule volume. Consequently, larger microcapsule diameters result in greater ohmic losses, increased electromagnetic energy absorption, and consequently higher electromagnetic shielding effectiveness (*SE*) values. The silicone matrix contains numerous dipoles that, when subjected to reflected electromagnetic waves in the dielectric layer, become repeatedly reoriented and polarized. This dipole excitation generates significant polarization losses [57,58] when the dipole reorientation lags behind the alternating electric field, thereby dissipating electromagnetic wave energy through

(7) $$W_{p}=DE^{2}f\eta$$

In the equation, *W_p_* represents the power dissipated as heat through dielectric polarization losses in the dielectric layer, *D* is a coefficient related to the dielectric layer’s structure, *E* denotes the internal electric field strength within the dielectric layer, and *η* signifies the electromagnetic energy dissipation factor. In this study, when incorporating numerous PUF-LM microcapsules to form microcapacitor structures with the silicone matrix dielectric layer, the repeated reflection and scattering of electromagnetic waves at the electrode-dielectric interfaces enhance both *W*_0_ and *W_p_*, thereby contributing to the overall electromagnetic shielding effectiveness (*SE*). The microcapacitor model treats the electrode plates themselves as resistors and combines them with the dielectric layer as a capacitor. Consequently, electron oscillations within the electrodes and dipole polarization in the dielectric layer collectively facilitate both reflection and absorption of electromagnetic waves [59]. The electromagnetic shielding performance can be improved through two primary approaches: (1) increasing ohmic losses by enlarging microcapsule volume, and (2) enhancing polarization effects by constructing more microcapacitor units.

In this study, when the microcapsule size was fixed at 9.8 μm, increasing the loading content from 30 wt% to 60 wt% led to the formation of more microcapacitor structures, thereby enhancing the electromagnetic shielding effectiveness (*SE*). As the microcapsule size decreases, assuming random uniform dispersion in the silicone matrix, Supplementary Figure S3 simulations show that smaller particles increase the number of microcapsules per unit volume, generating more microcapacitors and thus a greater *SE* contribution. For composites with a fixed 60 wt% loading but varying microcapsule sizes, the SE exhibited a non-monotonic trend: an initial increase, followed by a decrease, and then a final increase. Specifically, when the particle size decreased from 10.6 μm to 9.8 μm, the *SE* rose from 5.49 dB to 6.50 dB due to the increased microcapacitor density per unit volume. Further reduction to 8.6 μm caused the *SE* to decline to 5.91 dB, as particle agglomeration disrupted the ordered microcapacitor distribution, reducing the effective electrode area and ohmic losses. However, continued size reduction to 7.6 μm increased the *SE* to 7.48 dB, driven by enhanced interfacial polarization resulting from the greater specific surface area. In this case, the dominant effect of increased electrode–dielectric interfaces overcame the negative impact of partial agglomeration.

These results reveal the synergistic effects of microcapsule volume fraction and particle size on the electromagnetic shielding effectiveness (EMSE) of LMMCs. The volume fraction governs the linear increase in EMSE: when the particle size remains constant, the EMSE monotonically increases with microcapsule content due to the construction of additional microcapacitor structures. In contrast, particle size regulation leads to nonlinear changes in EMSE. When the microcapsule content is held constant, decreasing particle size triggers a non-monotonic EMSE response characterized by an “increase–decrease–reincrease” pattern, reflecting the competitive mechanism between microcapacitor density and dispersibility. Between 10.6 μm and 9.8 μm, the increase in microcapsule density dominates the EMSE enhancement. Between 9.8 μm and 8.6 μm, microcapsule aggregation reduces the EMSE. Between 8.6 μm and 7.6 μm, the high specific surface area boosts the EMSE again. Therefore, optimized design requires coordinated regulation of both the volume fraction and particle size of the microcapsules, along with suppression of microcapsule aggregation, to maximize the EMSE of the composite material.

**Figure 12:**
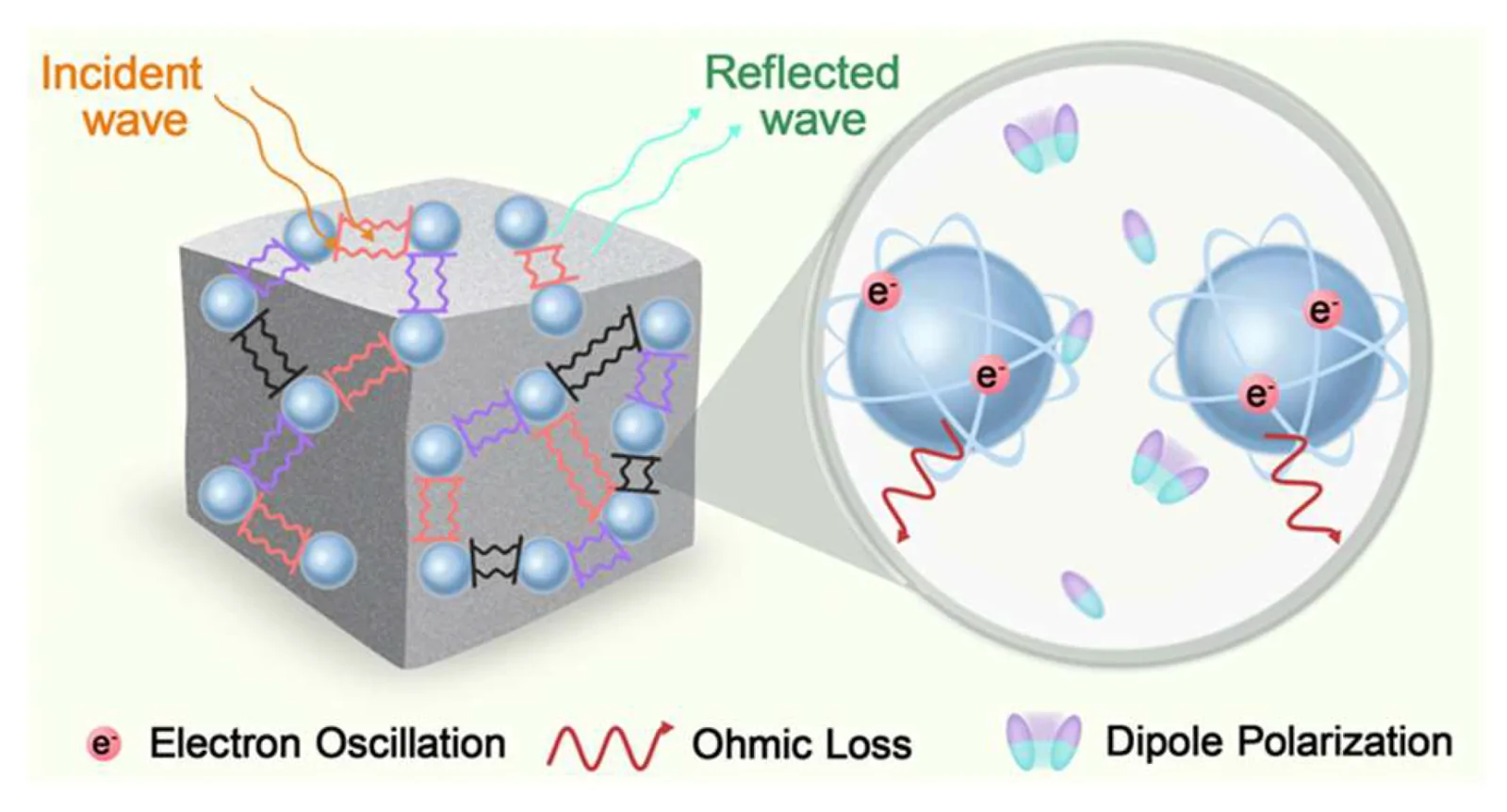
Schematic diagram of electromagnetic shielding of microcapacitors.

To further elucidate the EMI shielding mechanism, a microcapacitor model is proposed, as illustrated in Figure 12. In the LMMC structure, adjacent PUF-LM microcapsules are separated by the insulating PUF shell and the silicone matrix, forming numerous microscale capacitors. Under an incident electromagnetic wave, these microcapacitors undergo interfacial polarization (Maxwell–Wagner polarization) at the interfaces between the conductive liquid metal core and the insulating shell, as well as between the shell and the matrix. This polarization generates displacement currents and dielectric losses that attenuate the electromagnetic energy. The density of such microcapacitors increases with decreasing microcapsule size due to the higher number of particles per unit volume, which would intuitively enhance the *SE*. However, as observed experimentally, the *SE* does not monotonically increase with decreasing size. This can be explained by the competing effect of agglomeration: when microcapsules become too small (e.g., 8.6 μm), agglomeration reduces the uniformity of inter-particle spacing and introduces voids, which disrupts the ideal microcapacitor array and diminishes the polarization contribution. The subsequent recovery of *SE* at 7.6 μm arises from a further increase in microcapacitor density that partially compensates for the agglomeration effect. Thus, the proposed microcapacitor model provides a consistent qualitative explanation for the non-monotonic size dependence of EMI *SE*. Quantitative validation, including impedance spectroscopy and theoretical calculation of effective permittivity, is planned for future investigations.

## 3. Discussion

In this study, four types of PUF-LM microcapsules with distinct particle sizes (10.6 μm, 9.8 μm, 8.6 μm, and 7.6 μm) were successfully synthesized via in situ polymerization by adjusting the stirring rate and ultrasonic power. These core–shell microcapsules were subsequently blended with silicone rubber and freeze-dried to fabricate LMMCs with varying particle sizes and filler contents, yielding a novel insulating composite with electromagnetic shielding capability. Compared with the unfilled silicone matrix, the LMMCs exhibit notably improved thermal conductivity, reaching a maximum of 0.53 W·m^−1^·K^−1^ at 9.8 μm and 60 wt% loading, representing a 139.6% enhancement. This improvement benefits heat dissipation performance. Simultaneously, the composites maintain high electrical resistivity, with a maximum value of 8.99 × 10^11^ Ω·cm at 7.6 μm and 60 wt% loading, and a minimum of 3.63 × 10^11^ Ω·cm across all formulations. These values fully satisfy the insulation requirements for electronic packaging materials, effectively avoiding the short-circuit and leakage risks associated with conventional conductive fillers.

In addition to their insulating and thermally conductive properties, the LMMCs exhibit measurable electromagnetic shielding effectiveness (SE). At a fixed particle size of 9.8 μm, the SE reaches 7.29 dB at 60 wt% loading. The shielding mechanism is attributed to the microcapacitor structures formed within the composite, where the PUF-LM microcapsules act as electrode plates and the silicone matrix serves as the dielectric layer. Electron oscillations within the electrode plates and dipole polarization in the dielectric layer collectively contribute to the reflection and absorption of electromagnetic waves, thereby enhancing the SE. Both the particle size and the loading content of the microcapsules influence the shielding performance; increasing the microcapsule volume and content improves the SE. The proposed microcapacitor model provides a qualitative understanding of the EMI shielding mechanism. Future work should focus on quantitative validation through impedance spectroscopy and finite-element modeling to establish a direct correlation between microstructural parameters and shielding performance.

This material shows potential for further investigation in fields such as 5G communication, flexible electronics, aerospace, and medical equipment, particularly where both insulation and electromagnetic shielding are required. However, additional studies, including long-term reliability, leakage, mechanical fatigue, thermal cycling, and aging tests, are necessary to fully assess its suitability for practical applications, as the present work focuses on initial characterization under static conditions. Despite these remaining challenges, the current findings provide a foundation for the rational design of insulating composites with multifunctional capabilities.

## 4. Materials and Methods

### 4.1. Materials

The gallium-indium alloy (melting point: 16 °C, purity: 99.999%), urea (urea, 99%), formaldehyde solution (37 wt%, containing 10–15% methanol as a stabilizer), ammonium chloride (NH_4_Cl, 99.5%, Mw: 53.49), resorcinol (C_6_H_6_O_2_, Mw: 110.11, AR grade), poly(ethylene-alt-maleic anhydride) (average Mw: 100,000–500,000, powder), and sodium hydroxide solution (10 wt%, NaOH, for pH adjustment) were purchased from Shanghai Aladdin Biochemical Technology Co., Ltd., Shanghai, China. Deionized water (provided by the laboratory) and silicone adhesive (AB glue, SJ3211-A/SJ3211-B) were obtained from the Beijing Sanjing Xinde Technology company. All chemicals were used as received without further purification.

### 4.2. Preparation of PUF-LM Microcapsules

The schematic preparation process of PUF-LM microcapsules and their composite fabrication is presented in Figure 1. A 3 wt% poly(ethylene-alt-maleic anhydride) (EMA) solution was prepared in advance as a stock solution. Add 5 mL of EMA solution and 20 mL of deionized water to a 100 mL beaker; the mixture was homogenized using magnetic stirring. Subsequently, 0.05 g resorcinol, 0.065 g NaCl, and 0.5 g urea were sequentially added and stirred until complete dissolution. The pH of the solution was adjusted to 3.5 using NaOH solution (10 wt%). The pH-adjusted solution was transferred to a constant-temperature water bath set at 35 °C. Magnetic stirring was maintained at 1200 rpm, while ultrasonic power was varied (320 W and 400 W in initial trials, later fixed at 480 W) 27.34 g of gallium-indium alloy was slowly introduced into the solution under continuous ultrasonication and stirring (50 min). After 50 min, add 1.456 g of formaldehyde solution into the beaker. Turn off the ultrasonic device, adjust the water temperature to 60 °C, and end the reaction after 4.5 h of reaction. Cool the reactants to room temperature at room temperature. Wash and filter the reactants with deionized water repeatedly three times. Then, wash them repeatedly with absolute ethanol three times. Finally, wash them repeatedly with deionized water three times to obtain the microcapsules. After the washing is completed, put the microcapsules into a freeze dryer for freeze-drying, and finally obtain the PUF-LM microcapsule powder. Four groups of PUF-LM microcapsules with different particle sizes were synthesized by varying the ultrasonic power and stirring speed during the emulsification stage. Specifically, at a fixed stirring speed of 1200 rpm, ultrasonic powers of 320 W and 400 W were applied to obtain samples with average particle sizes of 10.6 μm and 9.8 μm, respectively. Then, at a fixed ultrasonic power of 480 W, stirring speeds of 800 rpm and 1600 rpm were used to obtain samples with average particle sizes of 8.6 μm and 7.6 μm, respectively. The particle size distribution of the microcapsules was determined using a Mastersizer 2000 laser diffraction particle size analyzer (Malvern Panalytical Ltd., Worcestershire, UK).

### 4.3. Preparation of LMMCs

The four types of as-prepared PUF-LM microcapsule powders were mixed with silicone rubber at mass fractions of 30 wt%, 40 wt%, 50 wt%, and 60 wt%, respectively. The mixtures were first homogenized in a beaker through mechanical stirring, then transferred into circular molds. To eliminate air bubbles trapped in the silicone matrix, the samples were stored in a freezer at −20 °C for 3 h. Finally, the composites were cured at room temperature to obtain the final LMMC samples.

### 4.4. Characterizations

The morphology and microstructure of the PUF-LM microcapsules and their dispersion within the silicone matrix were examined using scanning electron microscopy (SEM, Sigma 360, Carl Zeiss AG, Jena, Germany) at an accelerating voltage of 10 kV. Elemental composition and distribution were analyzed by integrated energy-dispersive X-ray spectroscopy (EDS). The average particle size of the microcapsules prepared under different conditions was determined using a laser particle size analyzer (Mastersizer 2000, Malvern Instruments Ltd., Malvern, Worcestershire, UK). Crystal structure and composition were characterized by powder X-ray diffraction (XRD) over a 2θ range of 10° to 90° at a scanning rate of 10°/min.

The thermal conductivity of LMMCs with varying microcapsule sizes and loading contents was measured using a thermal constant analyzer (TPS2500S, Hot Disk AB, Gothenburg, Sweden). Electrical resistivity was determined using a variable-temperature resistivity measurement system (RMS). Electromagnetic interference shielding effectiveness (EMI SE) was characterized in the X-band frequency range (8–12 GHz) using a vector network analyzer (E5071C, Keysight Technologies, Santa Rosa, CA, USA). For each composition, one sample was prepared and measured three times; the average value was reported.

## Data Availability

Data is contained within the article or Supplementary Materials.

## Supplementary Materials

The following supporting information can be downloaded at https://www.mdpi.com/article/10.3390/molecules31173009/s1. Figure S1: SEM images of liquid metal microcapsules with different particle sizes; Figure S2: SEM images and corresponding EDS elemental mappings (C, O, Ga, In) of liquid metal microcapsules with different particle sizes; Figure S3: Simulation of the distribution of different particle sizes in the same matrix; Figure S4: Cross-sectional SEM images of PUF-LM composites with different addition contents of 9.8 μm microcapsules; Figure S5: Cross-sectional SEM images and corresponding EDS elemental mappings (C, O, Ga, In) of LMMCs with 60 wt% microcapsules at different particle sizes.

## Abbreviations

The following abbreviations are used in this manuscript:

EMI	electromagnetic interference
PUF	poly(urea-formaldehyde)
LM	liquid metal
LMMCs	liquid metal microcapsule composites
EMC	electromagnetic compatibility
EMI SE	electromagnetic interference shielding effectiveness
TPU	thermoplastic polyurethane
MEPCM	microencapsulated phase change material
PUF-LM	poly(urea-formaldehyde)-encapsulated liquid metal

## References

[1] Han Y., Ruan K., Gu J. (2023). Multifunctional thermally conductive composite films based on fungal tree-like heterostructured silver nanowires@ boron nitride nanosheets and aramid nanofibers. Angew. Chem..

[2] Hudgins J.L. (2013). Power electronic devices in the future. IEEE J. Emerg. Sel. Top. Power Electron..

[3] Allendorf M.D., Schwartzberg A., Stavila V., Talin A.A. (2011). A roadmap to implementing metal-organic frameworks in electronic devices: Challenges and critical directions. Chem. Eur. J..

[4] Ahmed M., Peng M., Abana M., Yan S., Wang C. (2015). Interference coordination in heterogeneous small-cell networks: A coalition formation game approach. IEEE Syst. J..

[5] Song R., Jiang S., Hu Z., Fan C., Li P., Ge Q., Mao B., He D. (2022). Ultra-high conductive graphene assembled film for millimeter wave electromagnetic protection. Sci. Bull..

[6] Czepl N., Kircher D., Deutschmann B. Interference-Induced Electromagnetic Emission in Functioning Operating States of Integrated Circuits. Proceedings of the 2023 International Symposium on Electromagnetic Compatibility-EMC Europe.

[7] Pluska M., Czerwinski A., Ratajczak J., Kątcki J., Oskwarek Ł., Rak R. (2010). Identification of electron beam vibration sources by separation of magnetic distortion from electric distortion on scanning electron microscope imaging. J. Microsc..

[8] Kyechong K., Iliadis A.A. (2008). Latch-Up Effects in Cmos Inverters Due to High Power Pulsed Electromagnetic Interference. Solid-State Electron..

[9] Krupa I., Novák I., Chodák I. (2004). Electrically and Thermally Conductive Polyethylene/Graphite Composites and Their Mechanical Properties. Synth. Met..

[10] Shahzad F., Alhabeb M., Hatter C.B., Anasori B., Hong S.M., Koo C.M., Gogotsi Y. (2016). MATERIALS SCIENCE: Electromagnetic interference shielding with 2D transition metal carbides (MXenes). Science.

[11] Bo G., Ren L., Xu X., Du Y., Dou S. (2018). Recent progress on liquid metals and their applications. Adv. Phys. X.

[12] Chen S., Wang H.Z., Zhao R.Q., Rao W., Liu J. (2020). Liquid metal composites. Matter.

[13] Lu G., Ni E., Jiang Y., Wu W., Li H. (2024). Room-temperature liquid metals for flexible electronic devices. Small.

[14] Xu J. (2015). Liquid Metal Robotics: A New Category of Soft Robotics on the Horizon. Sci. Bull..

[15] Yan J., Lu Y., Chen G., Yang M., Gu Z. (2018). Advances in liquid metals for biomedical applications. Chem. Soc. Rev..

[16] Zhang W., Naidu B.S., Ou J.Z., O’Mullane A.P., Chrimes A.F., Carey B.J., Wang Y., Tang S.Y., Sivan V., Mitchell A. (2015). Liquid metal/metal oxide frameworks with incorporated Ga_2_O_3_ for photocatalysis. ACS Appl. Mater. Interfaces.

[17] Ralphs M.I., Kemme N., Vartak P.B., Joseph E., Tipnis S., Turnage S., Solanki K.N., Wang R.Y., Rykaczewski K. (2018). In situ alloying of thermally conductive polymer composites by combining liquid and solid metal microadditives. ACS Appl. Mater. Interfaces.

[18] Kong W., Wang Z., Wang M., Manning K.C., Uppal A., Green M.D., Wang R.Y., Rykaczewski K. (2019). Oxide-mediated formation of chemically stable tungsten-liquid metal mixtures for enhanced thermal interfaces. Adv. Mater..

[19] Liu S., Sweatman K., McDonald S., Nogita K. (2018). Ga-based alloys in microelectronic interconnects: A review. Materials.

[20] Zhao Z., Soni S., Lee T., Nijhuis C.A., Xiang D. (2023). Smart eutectic gallium-indium: From properties to applications. Adv. Mater..

[21] Duan Y., Yang W., Zhang Y., Gu Y., Xu P., Niu D., Huang Y., Song S., Puglia D., Ma P. (2025). Advanced hollow ball-cactus-like soft-magnetic LDH@ MXeneHT nanohybrid materials towards highly efficient electromagnetic protection. Compos. Part B Eng..

[22] Chen W., Hu X.N., Jiang J., Wang J.W., Guo J.L., Wu D., Zou J., Hu Z., Hu Q. (2025). Ultrahigh-strength Cu-10Fe-0.2 Mg in situ composites via ultra-nano precipitation within nanolamellar architectures. Vacuum.

[23] White S.R., Sottos N.R., Geubelle P.H., Moore J.S., Kessler M.R., Sriram S.R., Brown E.N., Viswanathan S. (2001). Autonomic healing of polymer composites. Nature.

[24] Lan X., Wang J., Chen L., Xu H., Zhang T., Chen Y. (2025). Spatially programmed regioisomeric conjugated microporous polymers modulating zinc sites for selective CO_2_ photoreduction to CH_4_. Chem. Sci..

[25] Visser J. (1989). Van der Waals and other cohesive forces affecting powder fluidization. Powder Technol..

[26] Werth J.H., Linsenbühler M., Dammer S.M., Farkas Z., Hinrichsen H., Wirth K.E., Wolf D.E. (2003). Agglomeration of charged nanopowders in suspensions. Powder Technol..

[27] Burger N., Laachachi A., Ferriol M., Lutz M., Toniazzo V., Ruch D. (2016). Review of thermal conductivity in composites: Mechanisms, parameters and theory. Prog. Polym. Sci..

[28] Zhiting T., Lee S., Chen G. (2014). A Comprehensive Review of Heat Transfer in Thermoelectric Materials and Devices. Annu. Rev. Heat Transf..

[29] Niu L., Liu Z., Liu G., Li M., Zong X., Wang D., An L., Qu D., Sun X., Wang X. (2022). Surface hydrophobic modification enhanced catalytic performance of electrochemical nitrogen reduction reaction. Nano Res..

[30] Sun B., Sun S., He P., Mi H.Y., Dong B., Liu C., Shen C. (2021). Asymmetric layered structural design with segregated conductive network for absorption-dominated high-performance electromagnetic interference shielding. Chem. Eng. J..

[31] Peterson R.A. (1994). A Meta-Analysis of Cronbach’s Coefficient Alpha. J. Consum. Res..

[32] Jeong C., Datta S., Lundstrom M. (2011). Full Dispersion Versus Debye Model Evaluation of Lattice Thermal Conductivity with a Landauer Approach. J. Appl. Phys..

[33] Malekpour H., Chang K.H., Chen J.C., Lu C.Y., Nika D.L., Novoselov K.S., Balandin A.A. (2014). Thermal conductivity of graphene laminate. Nano Lett..

[34] Zhai S., Zhang P., Xian Y., Zeng J., Shi B. (2018). Effective thermal conductivity of polymer composites: Theoretical models and simulation models. Int. J. Heat Mass Transf..

[35] Zhang J., You W., Wang Y., Yu W. (2026). Filler network breakdown triggered crack propagation in polymer nanocomposites. Compos. Part B Eng..

[36] Lai L., Niu B., Bi Y., Li Y., Yang Z. (2023). Advancements in SiC-reinforced metal matrix composites for high-performance electronic packaging: A review of thermo-mechanical properties and future trends. Micromachines.

[37] Su Z., Wang H., Ye X., Tian K., Huang W., He J., Guo Y., Tian X. (2018). Synergistic enhancement of anisotropic thermal transport flexible polymer composites filled with multi-layer graphene (mG) and mussel-inspiring modified hexagonal boron nitride (h-BN). Compos. Part A Appl. Sci. Manuf..

[38] Matzui L.Y., Syvolozhskyi O.A., Vovchenko L.L., Yakovenko O.S., Lazarenko O.A., Len T.A., Ischenko O.V., Dyachenko A.G., Vakaliuk A.V., Oliynyk V.V. (2022). Electrical and electromagnetic interference shielding properties of GNP-NiFe hybrid composite with segregate structure of conductive networks. J. Appl. Phys..

[39] Sumita M., Sakata K., Asai S., Miyasaka K., Nakagawa H. (1991). Dispersion of fillers and the electrical conductivity of polymer blends filled with carbon black. Polym. Bull..

[40] Tripathi M., Rahamtullah, Kumar D., Rajagopal C., Roy K.P. (2014). Influence of microcapsule shell material on the mechanical behavior of epoxy composites for self-healing applications. J. Appl. Polym. Sci..

[41] Balberg I. (1987). Tunneling and Nonuniversal Conductivity in Composite Materials. Phys. Rev. Lett..

[42] Vazquez A. (2025). Percolation in Higher Order Networks via Mapping to Chygraphs. J. Complex Netw..

[43] Winey K.I., Takashi K., Minfang M. (2007). Improving Electrical Conductivity and Thermal Properties of Polymers by the Addition of Carbon Nanotubes as Fillers. MRS Bull..

[44] Wang K., Cheng Q., Hou W., Guo H., Wu X., Wang J., Li J., Liu Z., Wang L. (2024). Unlocking the charge-migration mechanism in S-Scheme junction for photoreduction of diluted CO_2_ with high selectivity. Adv. Funct. Mater..

[45] Horibe H., Tomosumi K., Kunio Y. (2005). Electrical Conductivity of Polymer Composites Filled with Carbon Black. Jpn. J. Appl. Phys..

[46] Balasubramanian K.B.N., Ramesh T. (2018). Role, Effect, and Influences of Micro and Nano-Fillers on Various Properties of Polymer Matrix Composites for Microelectronics: A Review. Polym. Adv. Technol..

[47] Radzuan N.A.M., Zakaria M.Y., Sulong A.B., Sahari J. (2017). The effect of milled carbon fibre filler on electrical conductivity in highly conductive polymer composites. Compos. Part B Eng..

[48] Kunz K., Krause B., Kretzschmar B., Juhasz L., Kobsch O., Jenschke W., Ullrich M., Pötschke P. (2019). Direction dependent electrical conductivity of polymer/carbon filler composites. Polymers.

[49] Hassan T., Iqbal A., Yoo B., Jo J.Y., Cakmakci N., Naqvi S.M., Kim H., Jung S., Hussain N., Zafar U. (2024). Multifunctional MXene/carbon nanotube Janus film for electromagnetic shielding and infrared shielding/detection in harsh environments. Nano Micro Lett..

[50] Yun T., Kim H., Iqbal A., Cho Y.S., Lee G.S., Kim M.K., Kim S.J., Kim D., Gogotsi Y., Kim S.O. (2020). Electromagnetic shielding of monolayer MXene assemblies. Adv. Mater..

[51] Gao H., Wang Y.L., Xu W.L. (2025). Research Progress of Fiber-Based Electromagnetic Shielding Materials. Fibers Polym..

[52] Singh A.K., Shishkin A., Koppel T., Gupta N. (2018). A review of porous lightweight composite materials for electromagnetic interference shielding. Compos. Part B Eng..

[53] Liu L.P. (2010). Neutral Shells and Their Applications in the Design of Electromagnetic Shields. Proc. R. Soc. A Math. Phys. Eng. Sci..

[54] Iqbal A., Kwon J., Kim M.K., Koo C.M. (2021). Mxenes for Electromagnetic Interference shielding: Experimental and Theoretical Perspectives. Mater. Today Adv..

[55] Lv H., Yang Z., Liu B., Wu G., Lou Z., Fei B., Wu R. (2021). A flexible electromagnetic wave-electricity harvester. Nat. Commun..

[56] Iqbal A., Shahzad F., Hantanasirisakul K., Kim M.K., Kwon J., Hong J., Kim H., Kim D., Gogotsi Y., Koo C.M. (2020). Anomalous absorption of electromagnetic waves by 2D transition metal carbonitride Ti_3_CNTx (MXene). Science.

[57] Wang L., Ye Y., Chen N., Huang Y., Li L., Wu F., Chen R. (2018). Development and challenges of functional electrolytes for high-performance lithium-sulfur batteries. Adv. Funct. Mater..

[58] He M., Zhong X., Lu X., Hu J., Ruan K., Guo H., Zhang Y., Guo Y., Gu J. (2024). Excellent low-frequency microwave absorption and high thermal conductivity in polydimethylsiloxane composites endowed by hydrangea-like CoNi@BN heterostructure fillers. Adv. Mater..

[59] Zhou X., Min P., Liu Y., Jin M., Yu Z.Z., Zhang H.B. (2024). Insulating electromagnetic-shielding silicone compound enables direct potting electronics. Science.

