# Supplementary material for: Gallium-Based Liquid Metal Microcapsule Composites: A Synergistic Solution to the Challenges of Heat Dissipation, Electrical Insulation and Electromagnetic Shielding
Source: Molecules. 2026 Aug 27;31(17):3009. doi: 10.3390/molecules31173009 (PMC13567695; doi:10.3390/molecules31173009)
Supplement: Supplementary file 1 [file molecules-31-03009-s001.zip › molecules-4461301-supplementary.pdf]

Supporting information

Gallium-based liquid metal microcapsule composites: a synergistic solution to the challenges of heat dissipation, electrical insulation and electromagnetic shielding

Jie Bai [1, 2, 3], Cong Gu [1, 2, *], Shunfeng Sang [1], Zixin Zhang [1], Xiao Zhou [2], Junyu Liu [1], Shu Yang [1], Qiang Wang [1, 2, 3, *]

[1] Chongqing Key Laboratory of Green Aviation Energy and Power, Chongqing Jiaotong Universty, Chongqing 400074, P. R. China

[2] School of Mechatronics and Vehicle Engineering, Chongqing Jiaotong Universty, Chongqing 400074, P. R. China

[3] Key Laboratory of Low-grade Energy Utilization Technologies and Systems, Chongqing University, Ministry of Education, Chongqing 400044, P. R. China

* Corresponding author: Cong Gu and Qiang Wang

Email addresses: 622230990136@mails.cqjtu.edu.cn (C. Gu), wangq@cqjtu.edu.cn (Q. Wang)

Tel. / Fax: +86-023-47273962

Address: No.66 Xuefu Avenue, Nan 'an District, Chongqing 400074 P.R. China

Figure S1

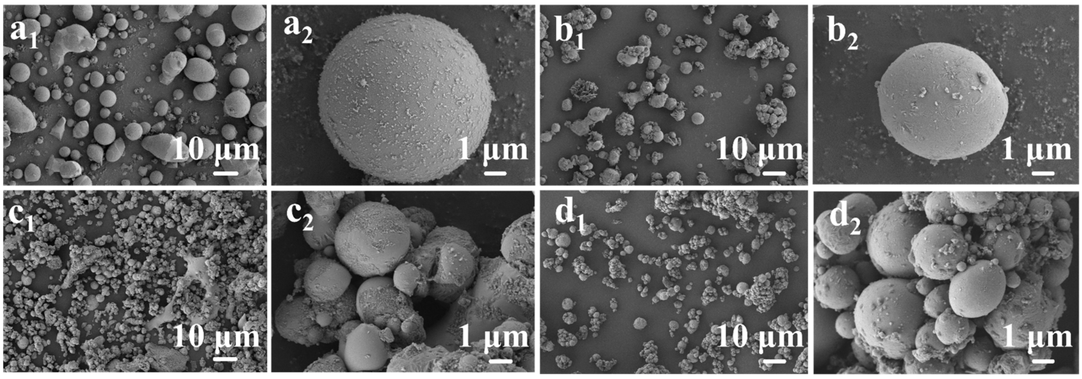

Figure S1. SEM images of liquid metal microcapsules with different particle sizes: (a_1-a_2) 10.6 μm, (b_1-b_2) 9.8 μm, (c_1-c_2) 8.6 μm, and (d_1-d_2) 7.6 μm.

This figure demonstrates the SEM images of different particle sizes, SEM images at magnifications of 10 μm and 1 μm are presented, where the particle sizes decrease sequentially from (a) to (d). As clearly observed in panels (a_2), (b_2), (c_2), and (d_2), the surfaces of the PUF-LM microcapsules are densely covered with submicron-sized particles, which are poly(urea-formaldehyde) polymer nanoparticles, which are linear PUF prepolymers formed by the polycondensation of methyl urea and amino group through a series of high-temperature reactions under alkaline conditions, and the different particle sizes of the PUF-LM microcapsules all had relatively intact surfaces without obvious ruptures or defects.

Figure S2

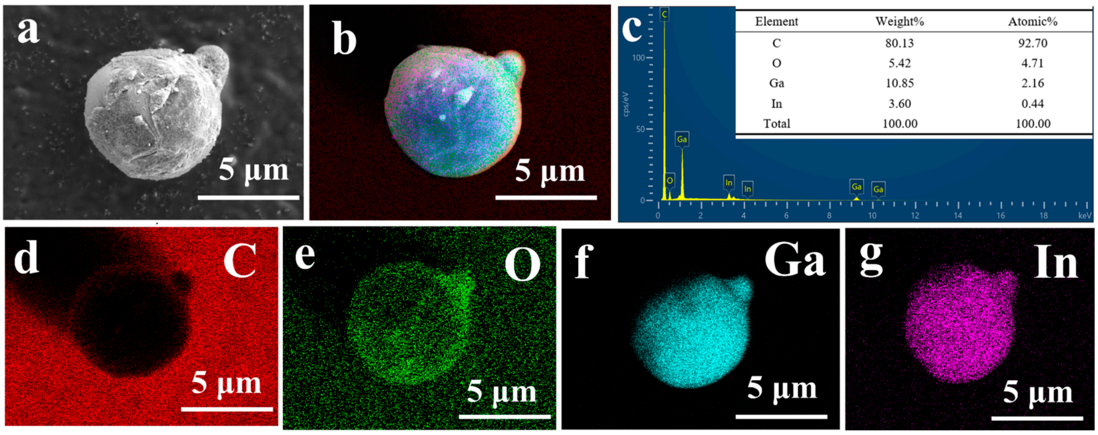

Figure S2-1. (a) SEM image of 9.8 μm microcapsules, (b) layered image, (c) total elemental spectrum of microcapsules, (d) C, (e) O, (f) Ga, (g) In.

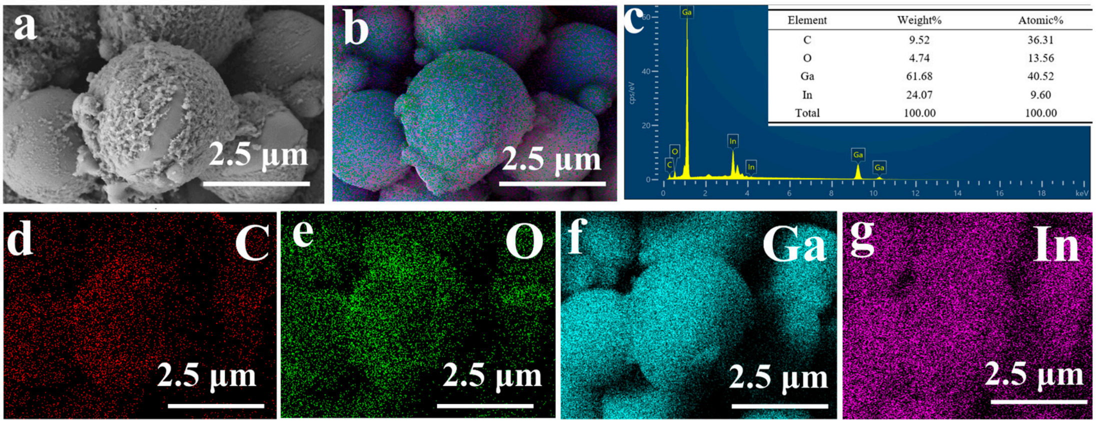

Figure S2-2. (a) SEM image of 8.6 μm microcapsules, (b) layered image, (c) total elemental spectrum of microcapsules, (d) C, (e) O, (f) Ga, (g) In.

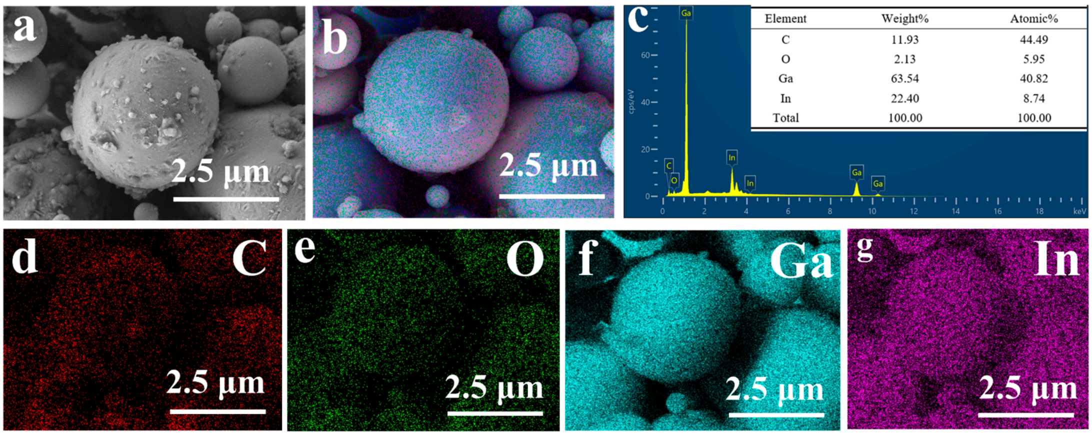

Figure S2-3. (a) SEM image of 7.6 μm microcapsules, (b) layered image, (c) total elemental spectrum of microcapsules, (d) C, (e) O, (f) Ga, (g) In.

Figure S2 presents the EDS spectra of PUF-LM microcapsules with diameters of 9.8 μm, 8.6 μm, and 7.6 μm, clearly revealing their elemental composition and distribution. Taking Figure S2-1 as an example, panel (c) demonstrates that the microcapsules primarily consist of four elements: C, O, Ga, and In. This confirms that the shell of the microcapsules is mainly composed of the organic polymer poly(urea-formaldehyde) (PUF), while the Ga and In elements originate from the liquid metal Ga-In alloy core. In contrast, the C and O elements are predominantly contributed by the PUF shell. The elemental mappings of C, O, Ga, and In (panels d, e, f, and g, respectively) exhibit consistent distribution patterns with the microcapsule morphology observed in panels (a) and (b).

Figure S3

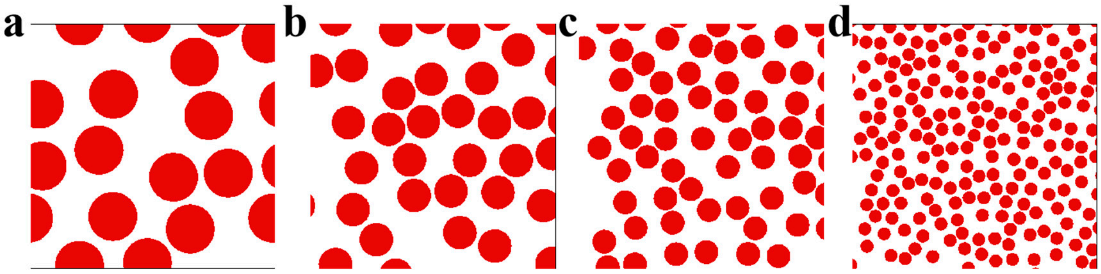

Figure S3. Simulation of the distribution of different particle sizes in the same matrix, (a)10.6 μm, (b)9.8 μm, (c)8.6 μm, (d)7.6 μm.

The simulated distribution of microcapsules with varying volumes within the same area is shown in the figure. From the simulations, it can be seen that as the particle size decreases, the number of microcapsules in the same matrix increases and it is easier to form a complete thermal conductivity network.

Figure S4

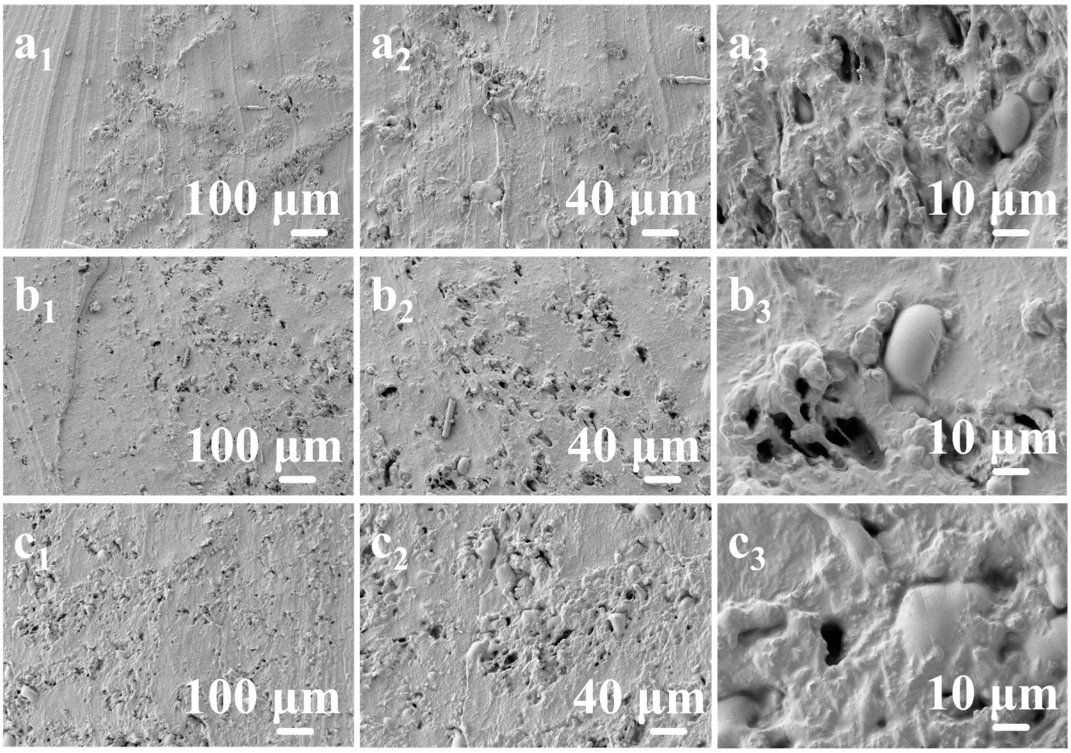

Figure S4. Cross-sectional SEM images of PUF-LM composites with different addition contents of 9.8μm microcapsules, (a_1-a_3) 30 wt%, (b_1-b_3) 40 wt%, (c_1-c_3) 50 wt%.

The cross-sections of LMMCs containing 9.8 μm microcapsules at different loading fractions (30 wt%, 40 wt%, 50 wt%, 60 wt%) were examined by SEM, as shown in Supplementary Material S5 (a_1-a_3: 30 wt%, b_1-b_3: 40 wt%, c_1-c_3: 50 wt%).As the amount of microcapsules added increases, the microcapsules are arranged more and more densely in LMMCS.

Figure S5

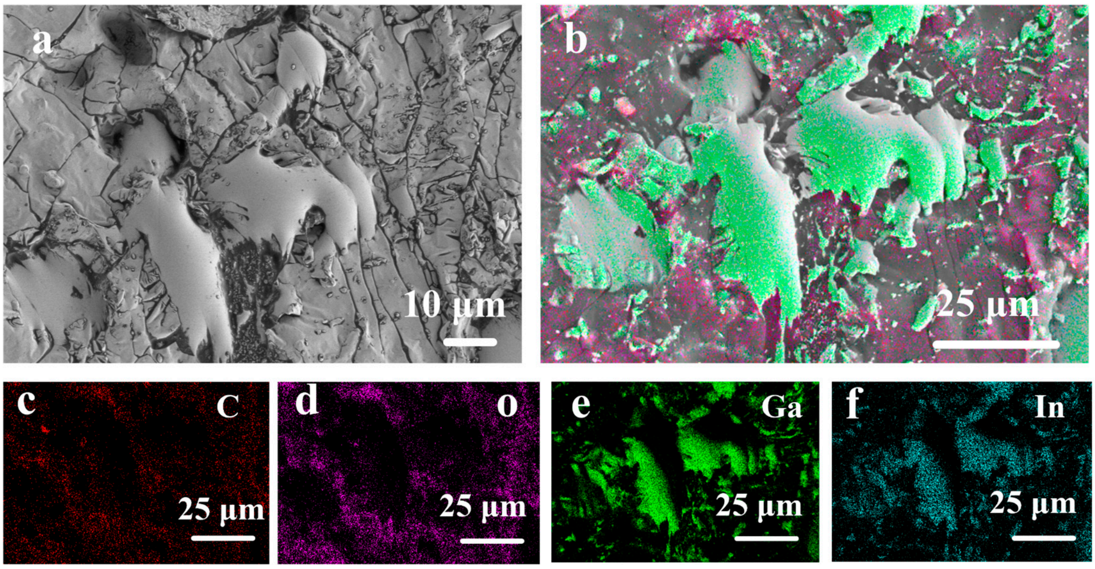

Figure S5-1. (a) Cross-sectional SEM image of 10.6 µm LMMCs with 60 wt% added microencapsulation content, (b) layered image, (c)C, (d)O, (e)Ga, (f)In.

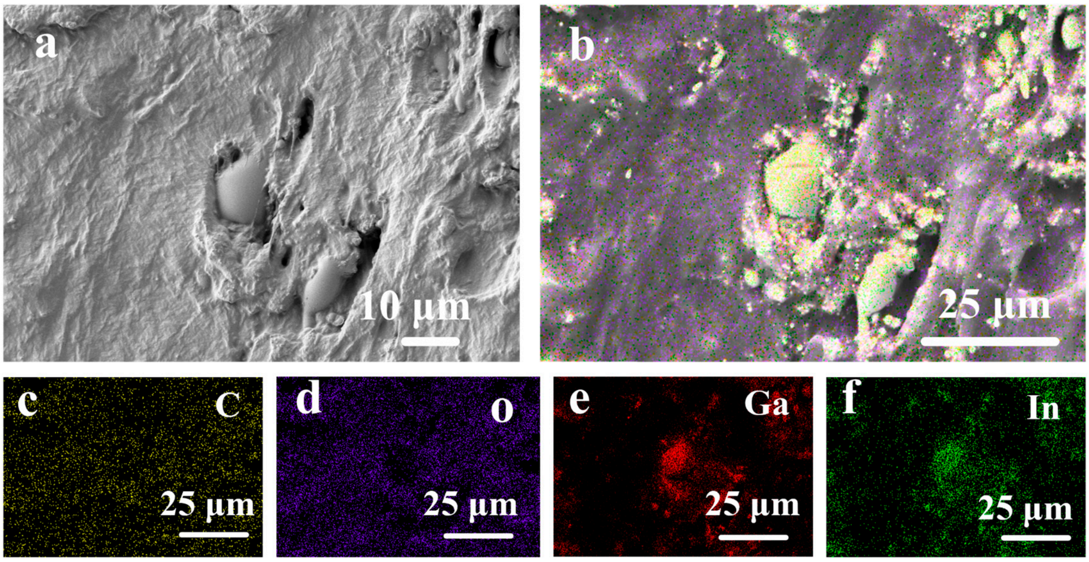

Figure S5-2. (a) Cross-sectional SEM image of 8.6 µm LMMCs with 60 wt% added microencapsulation content, (b) layered image, (c)C, (d)O, (e)Ga, (f)In.

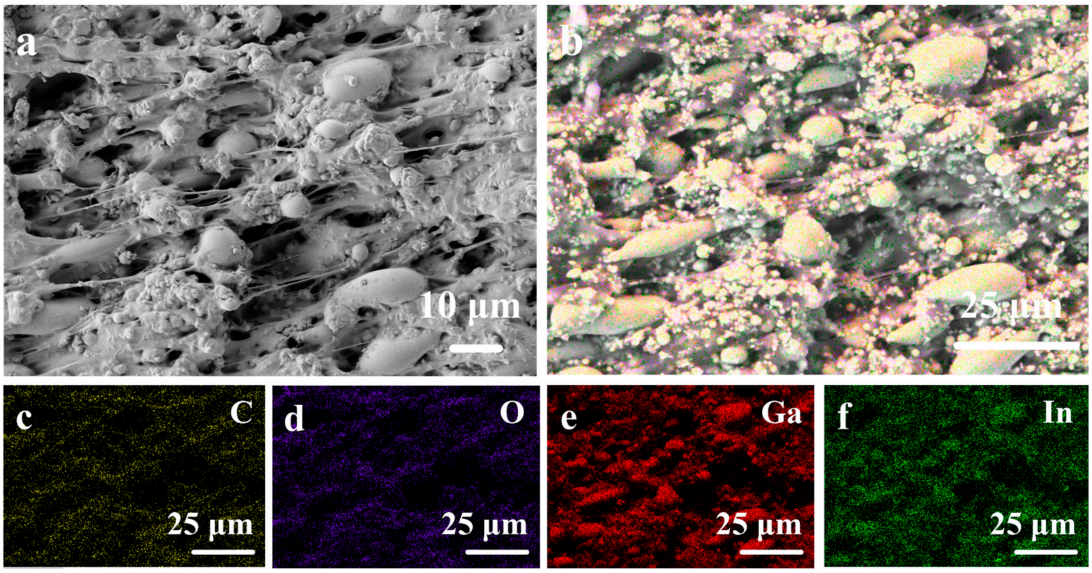

Figure S5-3. (a) Cross-sectional SEM image of 7.6 µm LMMCs with 60 wt% added microencapsulation content, (b) layered image, (c)C, (d)O, (e)Ga, (f)In.

Figure S5-1, S5-2, S5-3 shows SEM images of the cross sections of 10.6 µm, 8.6 µm, and 7.6 µm LMMCs when the addition amount of 60 wt% was constant, respectively. As observed in Figure S4-1(b), liquid metal leakage occurs during the cross-sectioning of larger LMMCs, likely due to microcapsule rupture. In contrast, this phenomenon is rarely observed in samples with diameters of 8.6 µm and 7.6 µm. EDS elemental analysis further confirms that the PUF-LM microcapsules are uniformly dispersed within the silicone matrix.
